# ER-luminal [Ca^2+^] regulation of InsP_3_ receptor gating mediated by an ER-luminal peripheral Ca^2+^-binding protein

**DOI:** 10.7554/eLife.53531

**Published:** 2020-05-18

**Authors:** Horia Vais, Min Wang, Karthik Mallilankaraman, Riley Payne, Chris McKennan, Jeffrey T Lock, Lynn A Spruce, Carly Fiest, Matthew Yan-lok Chan, Ian Parker, Steven H Seeholzer, J Kevin Foskett, Don-On Daniel Mak

**Affiliations:** 1 Department of Physiology, Perelman School of Medicine, University of PennsylvaniaPhiladelphiaUnited States; 2 Department of Statistics, University of PittsburghPittsburghUnited States; 3 Department of Neurobiology and Behavior, University of CaliforniaIrvineUnited States; 4 Proteomics Core Facility, The Children’s Hospital of PhiladelphiaPhiladelphiaUnited States; 5 Department of Physiology and Biophysics, University of CaliforniaIrvineUnited States; 6 Department of Cell and Developmental Biology, Perelman School of Medicine, University of PennsylvaniaPhiladelphiaUnited States; University of VermontUnited States; National Institute of Neurological Disorders and Stroke, National Institutes of HealthUnited States

**Keywords:** inositol 1,4,5-trisphosphate receptor, single-channel gating regulation, intracellular calcium-release channel, annexin, endoplasmic reticulum lumen, calcium signaling, None

## Abstract

Modulating cytoplasmic Ca^2+^ concentration ([Ca^2+^]_i_) by endoplasmic reticulum (ER)-localized inositol 1,4,5-trisphosphate receptor (InsP_3_R) Ca^2+^-release channels is a universal signaling pathway that regulates numerous cell-physiological processes. Whereas much is known regarding regulation of InsP_3_R activity by cytoplasmic ligands and processes, its regulation by ER-luminal Ca^2+^ concentration ([Ca^2+^]_ER_) is poorly understood and controversial. We discovered that the InsP_3_R is regulated by a peripheral membrane-associated ER-luminal protein that strongly inhibits the channel in the presence of high, physiological [Ca^2+^]_ER_. The widely-expressed Ca^2+^-binding protein annexin A1 (ANXA1) is present in the nuclear envelope lumen and, through interaction with a luminal region of the channel, can modify high-[Ca^2+^]_ER_ inhibition of InsP_3_R activity. Genetic knockdown of ANXA1 expression enhanced global and local elementary InsP_3_-mediated Ca^2+^ signaling events. Thus, [Ca^2+^]_ER_ is a major regulator of InsP_3_R channel activity and InsP_3_R-mediated [Ca^2+^]_i_ signaling in cells by controlling an interaction of the channel with a peripheral membrane-associated Ca^2+^-binding protein, likely ANXA1.

## Introduction

Modulating cytoplasmic free Ca^2+^ concentration ([Ca^2+^]_i_) is a universal signaling pathway that regulates numerous cell-physiological processes (Berridge, 2016). Ubiquitous endoplasmic reticulum (ER)-localized inositol 1,4,5-trisphosphate (InsP_3_) receptor (InsP_3_R) Ca^2+^-release channels play a central role in this pathway (Foskett et al., 2007). InsP_3_ generated in response to extracellular stimuli binds to and activates InsP_3_R channels to release Ca^2+^ stored in the ER lumen, generating diverse local and global [Ca^2+^]_i_ signals (Berridge, 2016). Whereas much is known regarding regulation of InsP_3_R channel gating by multiple processes, including binding of cytoplasmic ligands (Ca^2+^, InsP_3_ and ATP^4–^), post-translational modifications, interactions with proteins, clustering and differential localization (Berridge, 2016), the regulation of InsP_3_R channel activity by Ca^2+^ concentration in the ER lumen ([Ca^2+^]_ER_) is poorly understood and controversial (see (Caroppo et al., 2003; Yamasaki-Mann and Parker, 2011) and references therein). InsP_3_R activity influences [Ca^2+^]_ER_, which is critical for many processes, including regulation of bioenergetics, protein biogenesis and folding, and Ca^2+^ signaling (Corbett and Michalak, 2000). [Ca^2+^]_ER_ dysregulation is associated with pathological conditions, including ER stress responses, diabetes, cardiac dysfunction, neurodegeneration, defective cell proliferation and cell death (Berridge, 2016). Mechanisms that link cellular processes with [Ca^2+^]_ER_, and the connections between [Ca^2+^]_ER_ dysregulation and disease pathogenesis are ill-defined and poorly understood (Mekahli et al., 2011).

**Figure 1.**
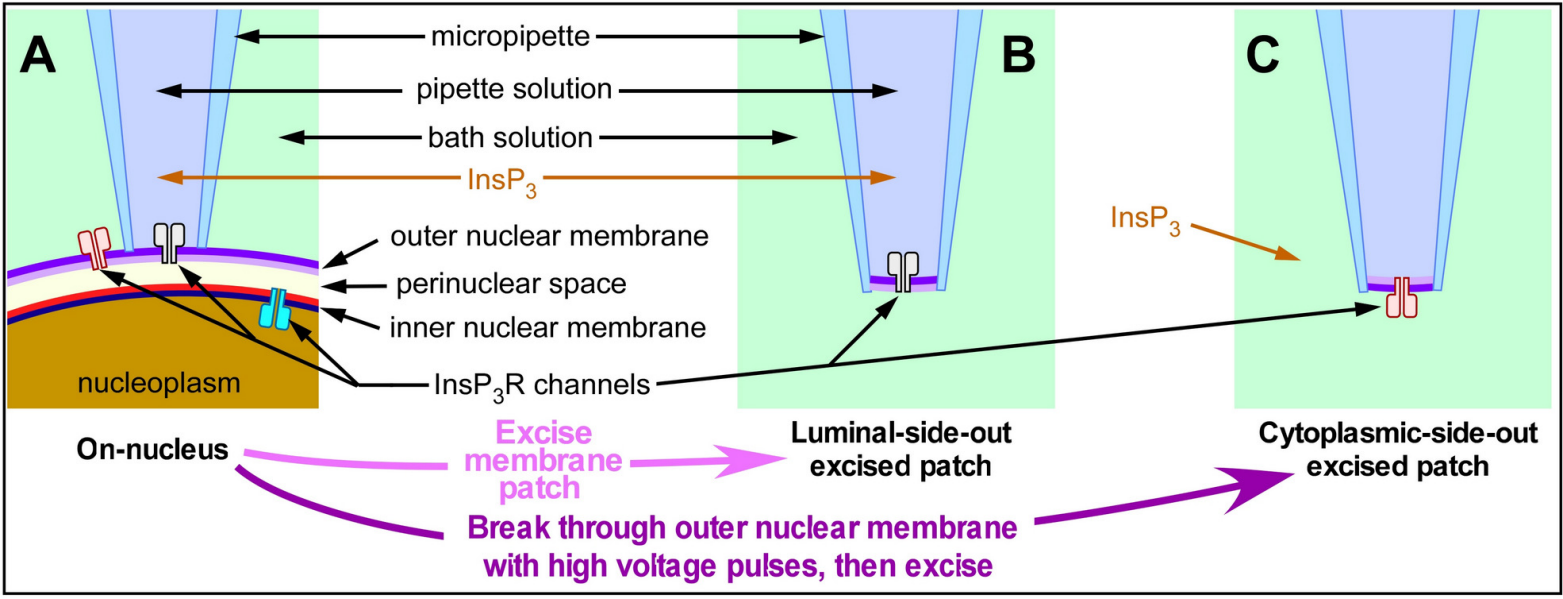
Schematic diagram illustrating the orientation of InsP_3_R channels in isolated nuclear membrane patches and InsP_3_-containing solution relative to the micropipette in various configurations of nuclear patch-clamping. (**A**) On-nucleus configuration with outer nuclear membrane intact, (**B**) excised luminal-side-out configuration, (**C**) excised cytoplasmic-side-out configuration.

For a long time, regulation of InsP_3_R channel activity by [Ca^2+^]_ER_ has primarily been examined by approaches that relied on changes in [Ca^2+^]_i_ or [Ca^2+^]_ER_ to infer channel activity (Caroppo et al., 2003; Yamasaki-Mann and Parker, 2011) and references therein), with no rigorous control of both [Ca^2+^]_ER_ and [Ca^2+^]_i_ simultaneously. It has therefore been difficult to differentiate feed-through effects in which Ca^2+^ flux through channel raises [Ca^2+^]_i_ at the cytoplasmic Ca^2+^-binding sites to modulate channel activity from direct effects of [Ca^2+^]_ER_ on the luminal aspect of the InsP_3_R. Patch-clamp electrophysiology allows rigorous, simultaneous control of [Ca^2+^] on both sides of the membrane, but intracellular ER membranes are not accessible to patch pipettes. To overcome this limitation, we made use of the fact that the ER membrane is continuous with the outer nuclear membrane (Dingwall and Laskey, 1992) and pioneered the use of nuclear patch-clamp electrophysiology on isolated nuclei (Mak et al., 2005). This enables the activities of single InsP_3_R channels to be recorded in their native ER membrane and luminal milieus. Different patch-clamp configurations have been used to study single InsP_3_R channels under rigorously controlled [Ca^2+^]_ER_ *and* [Ca^2+^]_i_: including on-nucleus (on-nuc, cytoplasmic aspect of the channel faces into the pipette solution with the luminal milieu intact, Figure 1A), excised luminal-side-out (lum-out, cytoplasmic aspect of the channel faces into the pipette solution with the luminal aspect exposed to the bath solution, Figure 1B) and excised cytoplasmic-side-out (cyto-out, cytoplasmic aspect of the channel perfused by the bath solution with the luminal aspect facing the pipette solution, Figure 1C; Mak et al., 2013a; Mak et al., 2013b). Because of the relatively low selectivity of InsP_3_R channels for Ca^2+^ vs K^+^ (15.2 : 1 [Vais et al., 2010a]) and orders of magnitude higher [K^+^] (140 mM) than that of other ions in the physiological solutions used in our experiments (70 nM to ≤600 μM [Ca^2+^]_free_; 0 or 200 μM [Mg^2+^]_free_), the electrical currents through open InsP_3_R channels are overwhelmingly carried by K^+^ in all our patch-clamp electrophysiology experiments, enabling the kinetics of channel gating to be studied with both luminal and cytoplasmic [Ca^2+^] well-defined and controlled.

**Figure 2.**
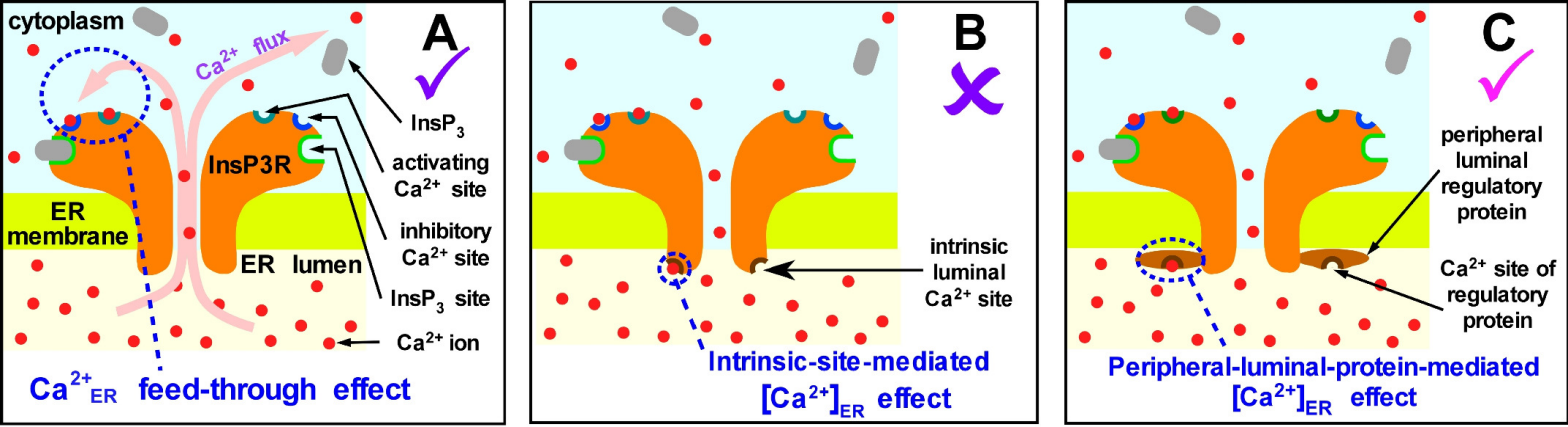
Possible mechanisms of [Ca^2+^]_ER_ regulation of InsP_3_R channel activity. (**A**) Ca^2+^ flux through an open InsP_3_R channel driven by high [Ca^2+^]_ER_ raises local [Ca^2+^]_i_ in the pore vicinity to regulate the channel through its cytoplasmic activating and inhibitory Ca^2+^-binding sites. (**B**) Ca^2+^ binds directly to an intrinsic site on the luminal side of the channel to regulate its activity. (**C**) Ca^2+^ binding to a peripheral protein in the ER lumen regulates channel activity indirectly, by promoting interaction of the peripheral protein with the InsP_3_R.

Using the nuclear patch-clamp approach in a previous study (Vais et al., 2012), we demonstrated that InsP_3_R channel activity can be modulated by [Ca^2+^]_ER_ indirectly via feed-through effects of Ca^2+^ flux driven through an open channel by high [Ca^2+^]_ER_ that raises the local [Ca^2+^]_i_ in the channel vicinity to regulate its activity through its cytoplasmic activating and inhibitory Ca^2+^-binding sites (Figure 2A). That study demonstrated that these feed-through effects can be completely abrogated by sufficient Ca^2+^ chelation on the cytoplasmic side to buffer local [Ca^2+^]_i_ at cytoplasmic Ca^2+^ binding sites of the InsP_3_R. In the presence of 5 mM 5,5’-diBromo BAPTA (a fast acting Ca^2+^ chelator) on the cytoplasmic side in the lum-out excised patch configuration (Figure 1B), the open probability *P*_o_ of the InsP_3_R channel did not change discernably when the [Ca^2+^]_ER_ was switched between 70 nM and 300 μM, no matter whether saturating 10 μM [InsP_3_] (Figure 2C in Vais et al., 2012) or sub-saturating 3 μM [InsP_3_] (Figure 7B in Vais et al., 2012) was present in the micropipette (2 μM [Ca^2+^]_i_ was in the micropipette in all experiments described here). Thus, as long as [Ca^2+^]_free_ on the cytoplasmic side was well buffered, switching [Ca^2+^]_ER_ between 70 nM and 300 μM in lum-out excised membrane patches had no effect on channel activity. This definitively ruled out the possibility that InsP_3_R activity is modulated directly by an intrinsic Ca^2+^-regulatory site on the luminal side that is either part of the InsP_3_R or of a protein that constitutively interacts with the channel (Figure 2B).

Notably, however, in all previous studies of InsP_3_R channel gating, nuclei were isolated into a bath solution with low [Ca^2+^]_free_ to simulate physiological resting [Ca^2+^]_i _(Mak and Foskett, 2015). Although sarco/endoplasmic reticulum Ca^2+^-ATPase (SERCA) is present in the nuclear envelope (Lanini et al., 1992), the bath solutions contained no MgATP to energize it. Thus, passive Ca^2+^ leaks equilibrated [Ca^2+^]_free_ in the perinuclear space of the isolated nuclei (effectively [Ca^2+^]_ER_) with bath [Ca^2+^] at unphysiologically low levels. Accordingly, possible [Ca^2+^]_ER_ regulation of channel activity by peripheral proteins in the lumen that interact with the InsP_3_R only in the presence of high, physiological [Ca^2+^]_ER_ (Figure 2C) would have been undetected in previous studies.

Here we specifically assessed whether such a peripheral luminal protein (PLP)-mediated [Ca^2+^]_ER_ regulation of InsP_3_R channel activity exists. We discovered a strong Ca^2+^ flux-independent inhibition of InsP_3_R channel activity mediated by accessory ER-luminal protein(s) only in the presence of high, physiological [Ca^2+^]_ER_. We identified the region of the InsP_3_R that is involved in this [Ca^2+^]_ER_-dependent inhibition, and discovered that the widely expressed Ca^2+^-binding protein annexin A1 (ANXA1) localizes not only to the cytoplasm but also to the perinuclear space inside the nuclear envelope, and possibly in the ER lumen as well, where it plays a critical role in channel inhibition through high [Ca^2+^]_ER_-mediated interaction with the InsP_3_R. Reducing this interaction enhanced agonist-induced InsP_3_R-mediated Ca^2+^ release, and increased local elementary Ca^2+^-release events in intact cells. Thus, [Ca^2+^]_ER_ is a major regulator of InsP_3_R-channel activity and InsP_3_R-mediated [Ca^2+^]_i_ signaling in cells by controlling an interaction of the channel with a luminal peripheral membrane-associated Ca^2+^-binding protein, likely ANXA1.

## Results

### Novel regulation by [Ca^2+^]_ER_ of InsP_3_R single-channel activity

**Figure 3.**
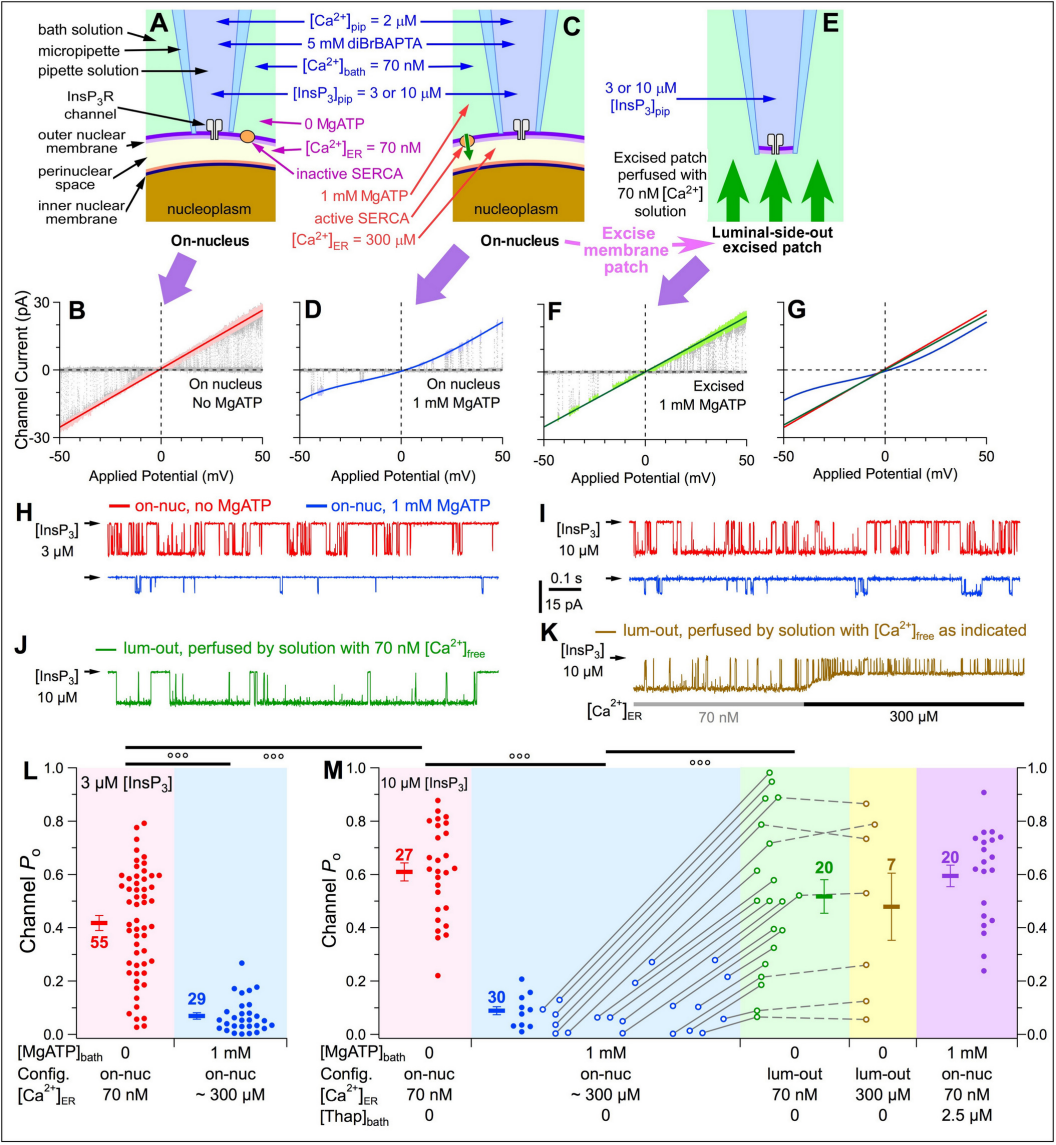
Raising [Ca^2+^]_ER_ in intact isolated nuclei affects activities of InsP_3_R channels. (**A and B**) On-nucleus (on-nuc) patch-clamp configuration and single-channel current-voltage (*i*_ch_*-V*_app_) plot of InsP_3_R channels in bath solution with 0-MgATP so SERCA was not active and [Ca^2+^]_ER_ equilibrated with [Ca^2+^]_bath_. (**C and D**) On-nuc configuration and *i*_ch_*-V*_app_ plot of InsP_3_R channels in bath solution with 1 mM MgATP to activate SERCA to move Ca^2+^ from the bath into the perinuclear space, raising [Ca^2+^]_ER_ to ~300 μM. ([Ca^2+^]_ER_ was estimated from the magnitude of the InsP_3_R channel current size [Vais et al., 2010a]). (**E and F**) Excised luminal-side-out (lum-out) configuration and *i*_ch_*-V*_app_ plot of InsP_3_R channels in isolated membrane patch perfused with 70 nM [Ca^2+^]_ER_ and 0-MgATP bath solution. The baseline closed-channel currents were subtracted from the *i*_ch_*-V*_app_ plots. Cytoplasmic [InsP_3_] in the pipette solution = 3 μM for sub-saturating level, and 10 μM for saturating level. *V*_app_ ramped from –50 to 50 mV (w.r.t. ground electrode in the bath) in 2 s. 10–20 ramps were averaged for each graph. Colored lines: linear (**B, F**) and 4^th^ order polynomial (**D**) fits to open-channel data points in graphs. In this and all subsequent patch-clamp experiments, optimal cytoplasmic [Ca^2+^]_free_ (2 μM) was used. In experiments shown here, [Ca^2+^]_free_ in bath solution = 70 nM. (**G**) Overlay of three fitted curves in (**B, D and F**). (**H**) Typical single-channel current traces recorded under constant *V*_app_ (–30 mV) in on-nuc configuration, with pipette solution containing sub-saturating 3 µM InsP_3_, and bath solutions containing 0- (red) or 1 mM-MgATP (blue). In this and all subsequent current traces, arrow on left of trace indicates closed-channel current level. (**I**) Corresponding current traces recorded with pipette solution containing saturating 10 µM InsP_3_. (**J**) Typical current trace recorded in lum-out configuration with constant *V*_app_ = –30 mV. (**K**) Typical current trace recorded as lum-out patch was perfused by solution with 70 nM Ca^2+^_free_ (grey bar) and then switched to one with 300 μM Ca^2+^_free_ (black bar). (**L**) *P*_o_ from individual current traces (filled circles), and their averages (thick horizontal bars) and s.e.m. (error bars) observed in on-nuc patch-clamp configuration with 3 µM InsP_3_ in pipette solution, and with 0- (red) or 1 mM (blue) MgATP in bath solutions. [Ca^2+^]_free_ on luminal side tabulated at *x*-axis. Numbers of current traces tabulated next to corresponding averages. In this and all subsequent data plots, symbols °, °° and °°° indicate *t*-test *p* value < 0.05, 0.005 and 0.001, respectively. (**M**) *P*_o_ from individual current traces (circles), averages (thick bars) and s.e.m. (error bars) observed with 10 µM InsP_3_ in pipette solution. Red symbols: *P*_o_ in on-nuc configuration with 0-MgATP in bath so [Ca^2+^]_ER_ = [Ca^2+^]_bath_ = 70 nM. Blue symbols: *P*_o_ in on-nuc configuration with 1 mM MgATP in bath so [Ca^2+^]_ER_ ~300 μM. Filled circles: *P*_o_ from experiments in which only the on-nuc configuration was achieved. Open circles: *P*_o_ from experiments in which lum-out configuration was achieved after on-nuc channel activity had been recorded. Green symbols: *P*_o_ observed in lum-out patches whose luminal side was perfused with solution containing 70 nM Ca^2+^_free_. Open circles connected with grey lines: *P*_o_ observed in same patch before and after membrane excision. Brown symbols: *P*_o_ observed in lum-out membrane patches after switching to perfusing solution containing 300 μM Ca^2+^_free_, as in (**K**). Open circles connected with dashed grey lines: *P*_o_ observed in same lum-out membrane patch before and after perfusion-solution switching. Purple symbols: *P*_o_ in on-nuc configuration with 1 mM bath MgATP and 2.5 μM thapsigargin.

We performed single-channel patch-clamp electrophysiology on outer membranes of nuclei isolated from mutant chicken B cells in which all three endogenous InsP_3_R genes were ablated and stably replaced with wild-type rat type-3 InsP_3_R (DT40-r3 cells) (Mak et al., 2005), so that homotetrameric rat type-3 InsP_3_R channels (Vais et al., 2012) were recorded in our experiments. In on-nuc patch-clamp experiments with the bath containing no MgATP, the [Ca^2+^]_ER_ in the perinuclear space (Figure 3A) equilibrated with [Ca^2+^]_bath_ (70 nM). Linear, ohmic InsP_3_R-channel current vs. applied potential (*i*_ch_-*V*_app_) curves were observed with high single-channel conductance, as expected (Figure 3B). In contrast, with 1 mM MgATP in the bath solution (Figure 3C), SERCA activity raised [Ca^2+^]_ER_ to physiological levels, if the outer and inner membranes of the isolated nuclei remained intact. The *i*_ch_-*V*_app_ curves (Figure 3D) observed were non-ohmic and asymmetric with respect to the origin, and single-channel conductance was reduced, both caused by permeant-ion (Ca^2+^) block of the measured K^+^ current due to high [Ca^2+^]_ER_. Thus, absence or presence of MgATP in the bath provides a mechanism to alter [Ca^2+^]_ER_ in intact nuclei. In a previous study examining the Ca^2+^ permeant-ion effect on K^+^ conductance through InsP_3_R channels (Vais et al., 2010a), we derived an empirical equation that describes, with high accuracy, the InsP_3_R channel slope conductance over a broad range of [Ca^2+^]_ER _(0 to 1.1 mM). This enabled us to use the single-channel conductance, rather than Ca^2+^ indicator dye fluorescence, to estimate that [Ca^2+^]_ER_ is ~300 μM in the perinuclear space of intact isolated DT40-r3 nuclei in the presence of bath MgATP. Subsequent excision of the membrane patch into the lum-out patch configuration (Mak et al., 2013c) exposed its luminal side to 70 nM [Ca^2+^]_bath_ (Figure 3E), which restored linear *I*_ch_-*V*_app_ curves and high single-channel conductance (Figure 3F).

**Figure 3—figure supplement 1.**
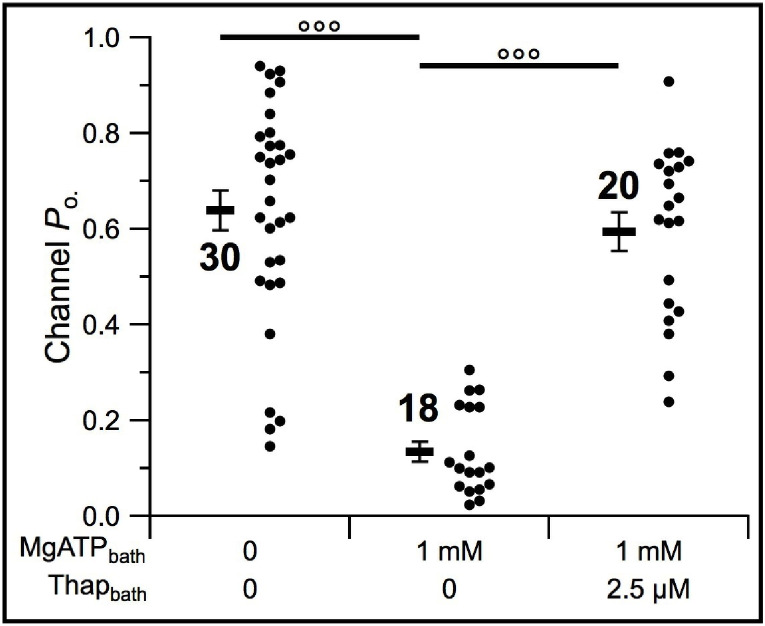
Thapsigargin abrogated SERCA activity despite the presence of bath MgATP. [Ca^2+^]_ER_ in intact isolated DT40-r3 nuclei equilibrated with [Ca^2+^]_bath_. InsP_3_R channel *P*_o_ of thapsigargin-treated nuclei were high, indistinguishable from *P*_o_ of nuclei exposed to no bath MgATP or thapsigargin. Channel *P*_o_ of individual current traces (circles) and their averages and s.e.m. (horizontal bars) are plotted for on-nuc patch-clamp experiments performed with bath solution containing 70 nM [Ca^2+^]_bath_. There is significant difference between *P*_o_ of channels in solutions with and without MgATP (both with no thapsigargin); and between *P*_o_ of channels in in solutions with and without thapsigargin (both with 1 mM MgATP). The controls (0 MgATP and 0 thapsigargin; 1 mM MgATP and 0 thapsigargin) and experiments (with thapsigargin and MgATP) were performed using cells harvested within at most one day apart to ensure that the qualities of the cells were similar.

In on-nuc patches of DT40-r3 nuclei, InsP_3_R channel open probability (*P*_o_) was high with 70 nM Ca^2+^_ER_ (Figure 3H and L, red) whereas it was profoundly reduced with [Ca^2+^]_ER_ raised to 300 μM by addition of MgATP to the bath (Figure 3H and L, blue). Even in saturating (10 μM) [InsP_3_], 300 μM Ca^2+^_ER_ strongly reduced *P*_o_ (Figure 3I and M, blue), to a level indistinguishable from that observed in 3 μM InsP_3_ (Figure 3L, blue). In contrast, when [Ca^2+^]_ER_ = 70 nM, *P*_o_ was higher in 10 μM InsP_3_ (Figure 3M, red) than in 3 μM InsP_3_ (Figure 3L, red). With the bath solution containing 1 mM MgATP and the SERCA inhibitor thapsigargin (2.5 μM) (Figure 3M, purple and Figure 3—figure supplement 1), *P*_o_ was indistinguishable from that in 0-MgATP_bath_, confirming that bath MgATP raised [Ca^2+^]_ER_ in intact nuclei by supporting SERCA activity. Because high concentrations of Ca^2+^ chelator (5 mM diBrBAPTA) buffered [Ca^2+^]_i_ at cytoplasmic Ca^2+^ regulatory sites, possible feed-through effects were eliminated (Vais et al., 2012). Thus, with an intact ER-luminal milieu and [Ca^2+^]_ER_ at a level characteristic of replete ER stores, InsP_3_R activity is profoundly suppressed by a mechanism unrelated to but as powerful as the well-known high-[Ca^2+^]_i_ inhibition of InsP_3_R activity. Because luminal [Ca^2+^] does not directly affect InsP_3_R channel activity (Vais et al., 2012), these results suggest a novel regulatory mechanism, possibly mediated by a resident luminal Ca^2+^-binding protein.

After recording channel activity in the on-nuc patch-clamp configuration, patches were excised into the lum-out configuration to observe gating of channels with their luminal sides exposed to bath [Ca^2+^]_free_ = 70 nM. Since [Ca^2+^]_i_ and [Ca^2+^]_ER_ experienced by channels in this configuration were effectively the same as those in the on-nuc configuration with no bath MgATP, it was expected that the inhibitory luminal-protein-mediated effect would be abrogated. In agreement, similar *P*_o_ were observed for channels in this lum-out configuration (Figure 3J and M, green) and channels in the on-nuc configuration in 0-MgATP (Figure 3I and M, red). Importantly, channel *P*_o_ remained high during subsequent perfusion with 300 μM Ca^2+^_free_ (Figure 3K and M, brown). Lack of inhibition when [Ca^2+^] was raised to 300 μM suggests that the luminal effector mediating high-[Ca^2+^]_ER_ inhibition is only loosely associated with the InsP_3_R, becoming irretrievably lost when the luminal side of the isolated patch was perfused by low-[Ca^2+^]_free_ solution.

Since the levels of suppression of InsP_3_R channel activities in the presence of high [Ca^2+^]_ER_ were independent of [InsP_3_] used (Figure 3L and M), all subsequent experiments were performed with saturating 10 μM [InsP_3_] to avoid possible ambiguous results due to insufficient stimulation of InsP_3_R channels.

**Figure 4.**
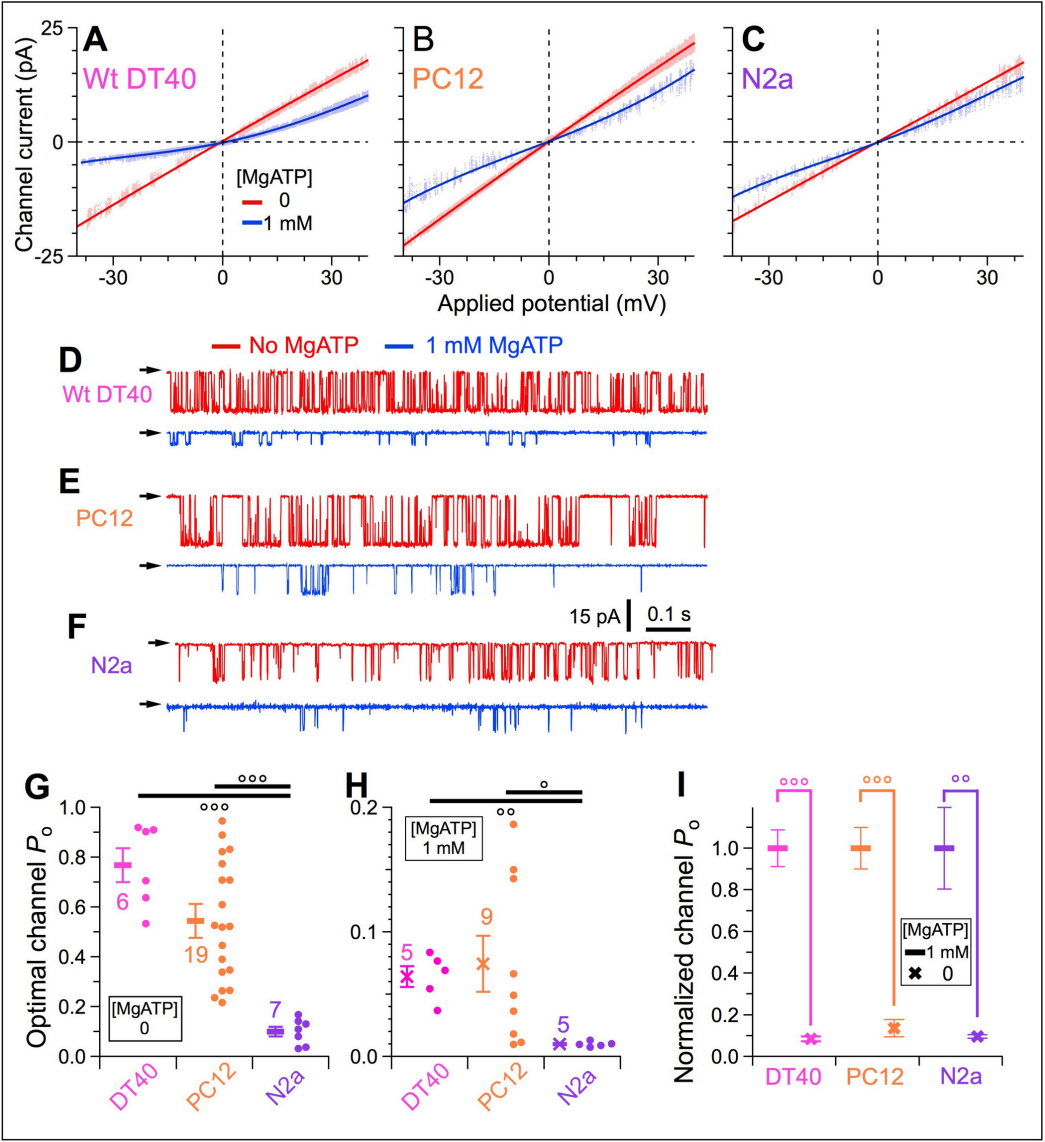
Activities of InsP_3_R channels from a number of cell lines expressing various InsP_3_R isoforms in different proportions are similarly affected by rise in [Ca^2+^]_ER_ in intact isolated nuclei. (**A–C**) Single-channel *I-V*_app_ plots of on-nuc patch-clamp experiments of endogenous InsP_3_R channels from WT DT40 (**A**), PC12 (**B**), and N2a (**C**) cells. Channels activated by 10 μM InsP_3_ with (blue) or without (red) 1 mM MgATP. Data obtained using protocol as in Figure 3 with *V*_app_ ramped from –40 to 40 mV in 2 s. Lines: linear (red) and 4^th^ order polynomial (blue) fits to open-channel data points. (**D***–***F**) Typical current traces for endogenous InsP_3_R channels from WT DT40 (**D**), PC12 (**E**), and N2a (**F**) cells, in presence (blue) and absence (red) of 1 mM MgATP in bath with *V*_app_ = –40 mV. (**G**) Optimal *P*_o_ activated by 10 μM InsP_3_ and 2 μM Ca^2+^_i_ in individual experiments (circles) and averages and s.e.m. (bars) for endogenous WT DT40 (magenta), PC12 (orange) and N2a (purple) InsP_3_R channels in bath without MgATP. (**H**) Optimal *P*_o_ in bath with 1 mM bath MgATP activated by the same ligand conditions as (**G**) for the same endogenous InsP_3_R channels. (**I**) Normalized *P*_o_, their averages and s.e.m. for endogenous InsP_3_R channels in bath with 1 mM MgATP, relative to respective average optimal *P*_o_ in (**G**).

To determine whether peripheral luminal-protein (PLP) inhibition affects endogenous InsP_3_R in different cell types, similar on-nuc patch-clamp experiments were conducted with intact nuclei (verified by asymmetric *i*_ch_-*V*_app_ curves [Figure 4A–C] and reduced single-channel conductance [Figure 4D–F]) isolated from mouse N2a cells, which predominantly express type 1 InsP_3_R (InsP_3_R–1) (Wojcikiewicz, 1995), rat PC-12 cells expressing mainly InsP_3_R-1 and InsP_3_R-3 (Newton et al., 1994), and wild-type (WT) chicken DT40 cells, which express all three isoforms (Sugawara et al., 1997). In a bath containing 0-MgATP, channels from the different cell types exhibited different maximum *P*_o_ (Figure 4D–F red traces, and Figure 4G). With high [Ca^2+^]_ER_ generated by 1 mM bath MgATP, *P*_o_ of the various endogenous InsP_3_R channels were strongly suppressed (Figure 4D–F blue traces, and Figure 4H). Interestingly, *P*_o_ of the different InsP_3_Rs were strongly suppressed to very similar extents (*P*_o_ reduction: WT DT40 92%, PC-12 86%, N2a 90% (Figure 4I), comparable to that of InsP_3_R-3 in DT40-r3 cells (85%) (Figure 3M, red and blue). This suggests that the inhibitory protein is present in the ER lumen of many cell types and interacts with all three InsP_3_R isoforms under high [Ca^2+^]_ER_.

### A luminal loop of the InsP_3_R is critical for luminal protein-mediated [Ca^2+^]_ER _regulation

**Figure 5.**
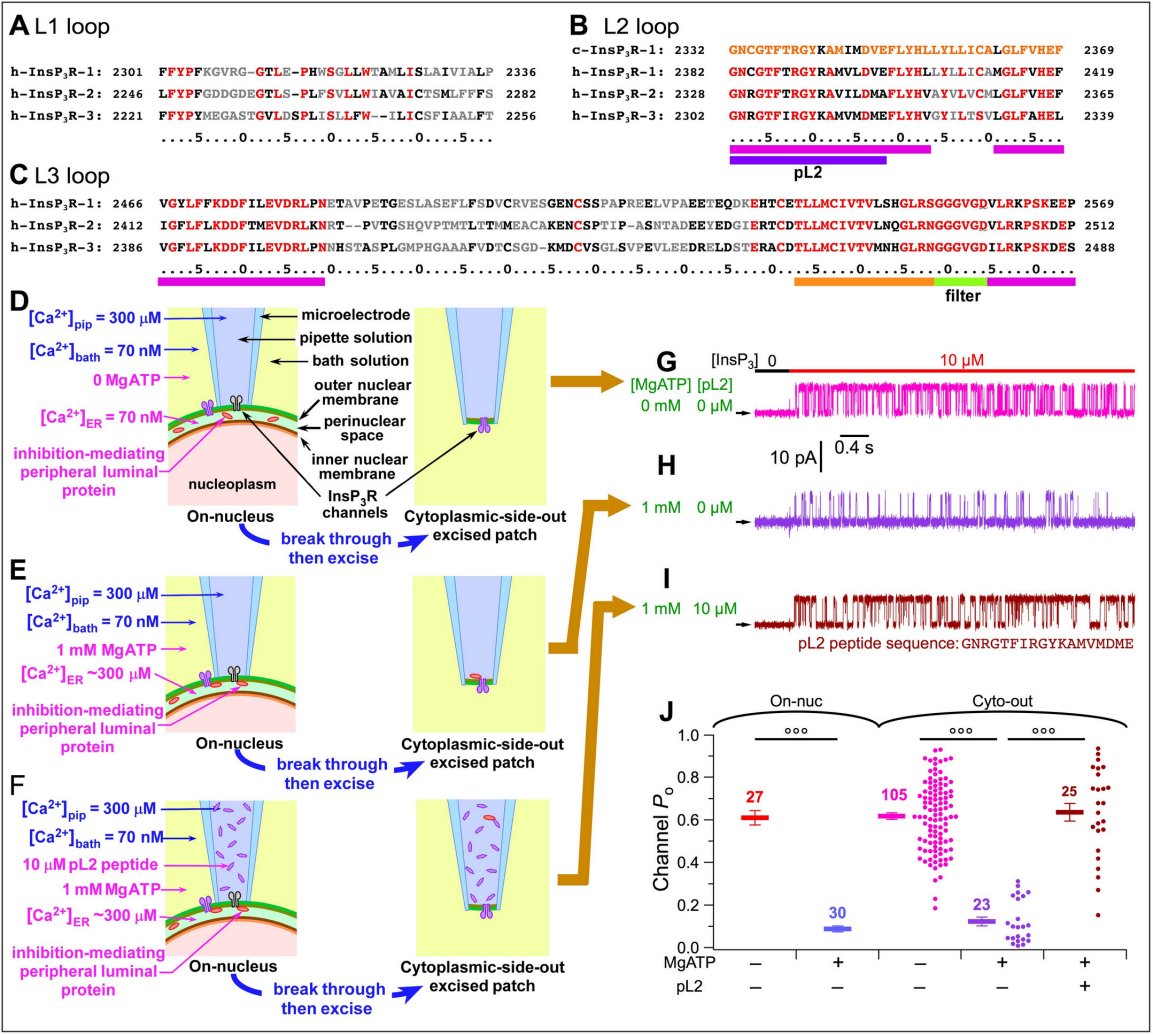
Identification of the InsP_3_R region involved in [Ca^2+^]_ER_ regulation of channel activity. (**A***–***C**) Sequences of L1 (**A**), L2 (**B**) and L3 (**C**) loops of human InsP_3_R isoforms exposed to ER lumen according to h-InsP_3_R-1 cryo-EM structure in Fan et al., 2015. Residues conserved over all three InsP_3_R isoforms are in red; similar residues in black; and different residues in grey. Highly-conserved sequences: magenta stripes. Sequence of pL2 (partial L2) peptide used in experiments is from h-InsP_3_R-3: purple stripe. L2 loop sequence of chicken InsP_3_R-1 also shown (*B*, top line) revealing highly-conserved L2 loop sequence. (**D***–***F**) Cartoons showing [Ca^2+^]_ER_-dependent interaction between luminal part of InsP_3_R and a peripheral protein in ER lumen in various pipette and bath solutions, during on-nuc or cyto-out nuclear patch-clamp experiments, as labeled. (**G***–***I**) Typical InsP_3_R-3 current traces in cyto-out experiments with 0- or 1 mM MgATP in bath, and 0- or 10 μM pL2 peptide in pipette solution, as tabulated. Traces in (**G**), (**H**) and (**I**) recorded in experiments depicted in (**D**), (**E**) and (**F**), respectively. In this and all subsequent cyto-out experiments, *V*_app_ = +30 mV and channels were activated by perfusion solutions containing optimal 2 μM Ca^2+^_i_, saturating 10 μM InsP_3_ and 0.5 mM ATP^4–^. (**J**) *P*_o_ of individual current traces (circles) and averages and s.e.m. (horizontal bars) observed under conditions as tabulated. For comparison, averages and s.e.m. of *P*_o_ in on-nuc experiments (red and blue horizontal bars) from Figure 3J are shown.

We hypothesized that the PLP interacts directly with the InsP_3_R. Only a small fraction of the InsP_3_R sequence is exposed to the ER lumen, consisting of three loops (L1, L2, and L3) connecting transmembrane helices in the pore-forming domain. We selected human sequences (Figure 5A–C) based on structural information about the InsP_3_R (Fan et al., 2015). Since the inhibitory effects were observed in cells expressing different channel isoforms, we selected sequences conserved in all three InsP_3_R isoforms. We excluded the P-region (orange stripe) and selectivity filter (green stripe) involved in other crucial channel functions (Figure 5C). Only four short sequences (magenta stripes in Figure 5B–C) fit the criteria to be luminal region(s) of the InsP_3_R that could interact with the PLP.

We first investigated whether the conserved region of the L2 loop (pL2, Figure 5B) is involved, by recording InsP_3_R-3 channels in the cytoplasmic-side-out (cyto-out) configuration (Mak et al., 2013b) with pipette solutions that faced the luminal aspect of the channel containing synthetic pL2 peptides. Nuclei were first exposed to [Ca^2+^]_bath_ = 70 nM and 0-MgATP, so [Ca^2+^]_ER_ equilibrated with [Ca^2+^]_bath _at 70 nM (Figure 5D, left) promoting dissociation of PLP from the InsP_3_R. When the cyto-out patch configuration was achieved (Figure 5D, right), the luminal aspect of the channel faced the pipette solution containing [Ca^2+^]_pip_ = 300 μM. Because the PLP had already dissociated from the channel before patch excision (Figure 5D, left), we expected no inhibition to be observed. Indeed, when the patch was perfused with saturating 10 μM InsP_3_ and optimal 2 μM Ca^2+^_i_ (buffered with 5 mM dibromoBAPTA) (Vais et al., 2010b), channels were activated with reduced conductance due to permeant Ca^2+^ block caused by high [Ca^2+^]_ER_ in the pipette solution, but they exhibited high *P*_o_ (~0.6) (Figure 5G and J, magenta) indistinguishable from that observed in on-nuc experiments under similar conditions (red bars in Figure 5J). Alternately, nuclei were first exposed to 1 mM MgATP in the bath to raise [Ca^2+^]_ER_ to ~300 μM to promote an interaction between the PLP and the InsP_3_R (Figure 5E, left). Because the pipette solution contained 300 μM Ca^2+^, the interaction remained intact when the cyto-out patch configuration was achieved (Figure 5E, right). Indeed, low channel *P*_o_ was observed with maximal stimulation (Figure 5H and J, purple), similar to the observations made using the on-nuc configuration (Figure 5J, blue bars). Having therefore established that the inhibitory effect of the PLP could be observed in the cyto-out patch configuration, we included 10 μM pL2 peptide in the pipette solution (Figure 5F, left). With pL2 peptides present on the cytoplasmic side in the on-nuc configuration before establishing the cyto-out configuration, InsP_3_R remained bound to the PLP before membrane excision. However, after achieving the cyto-out configuration, the luminal side of the InsP_3_R became exposed to the peptides. We reasoned that if the pL2 region is involved in the inhibitory effects through interactions with the PLP, then pL2 peptide in high concentrations should compete the PLP away from the InsP_3_R, and thereby raise *P*_o_ despite presence of 300 μM Ca^2+^ (Figure 5F, right). Indeed, inhibition was completely abolished such that the *P*_o_ of channels with pL2 peptides on their luminal side (Figure 5I–J, brown) was indistinguishable from *P*_o_ recorded in on-nuc (red) or cyto-out patch-clamp experiments with 0-MgATP in the bath (magenta). This indicates that the pL2 region interacts with the PLP to suppress InsP_3_R channel activity, and that the inhibitory effect of the PLP is mostly or completely mediated by the pL2-PLP interaction.

### Identification of the inhibitory peripheral protein(s) in the ER lumen

To identify the PLP, peptides with a modified pL2 sequence (mpL2) or scrambled mpL2 (smpL2) were used for in-vitro pull-downs from soluble fractions of bovine hepatocyte ER microsomes (Figure 6—figure supplement 1). Eluates from the pull-downs were analyzed by mass spectrometry. Among >460 proteins detected, we looked for those enriched in the mpL2, that are Ca^2+^-binding proteins (UniProt protein database) in the ER lumen (or in topologically-equivalent locations), and that are broadly expressed. The annexin (ANX) protein family met these criteria. Of 12 members of the ANX family, five were identified to have statistically-significant preference to bind to the mpL2 peptide over the smpL2 peptide (Figure 6—source data 1, also see ‘Ca^2+^-dependent affinity enrichment mass spectrometry’ in Materials and methods).

ANX proteins are ubiquitously expressed, Ca^2+^-dependent phospholipid-binding proteins (Raynal and Pollard, 1994). Ca^2+^ binding to ANX (dissociation constant *K*_d_ ~25 μM to 1 mM) promotes its association with negatively-charged phospholipid-containing membranes (Raynal and Pollard, 1994; Gerke and Moss, 2002). Although ANX proteins are known to be cytoplasmic, many studies have reported their presence in locations topologically equivalent to the ER lumen, including the extracellular side of the plasma membrane (PM) (Solito et al., 1994; Brownstein et al., 2001; McArthur et al., 2009; Yáñez et al., 2012; Mirkowska et al., 2013), within intracellular and secretory granules (McArthur et al., 2009; Leoni et al., 2015; Chi et al., 2006; Boudhraa et al., 2016; Perretti et al., 2000), and in the extracellular space (McArthur et al., 2009; Boudhraa et al., 2016; Arur et al., 2003; Fan et al., 2004; Scannell et al., 2007; D'Acquisto et al., 2007; Iwasa et al., 2012; Belvedere et al., 2014), suggesting that ANX can also be present in the ER lumen.

**Figure 6.**
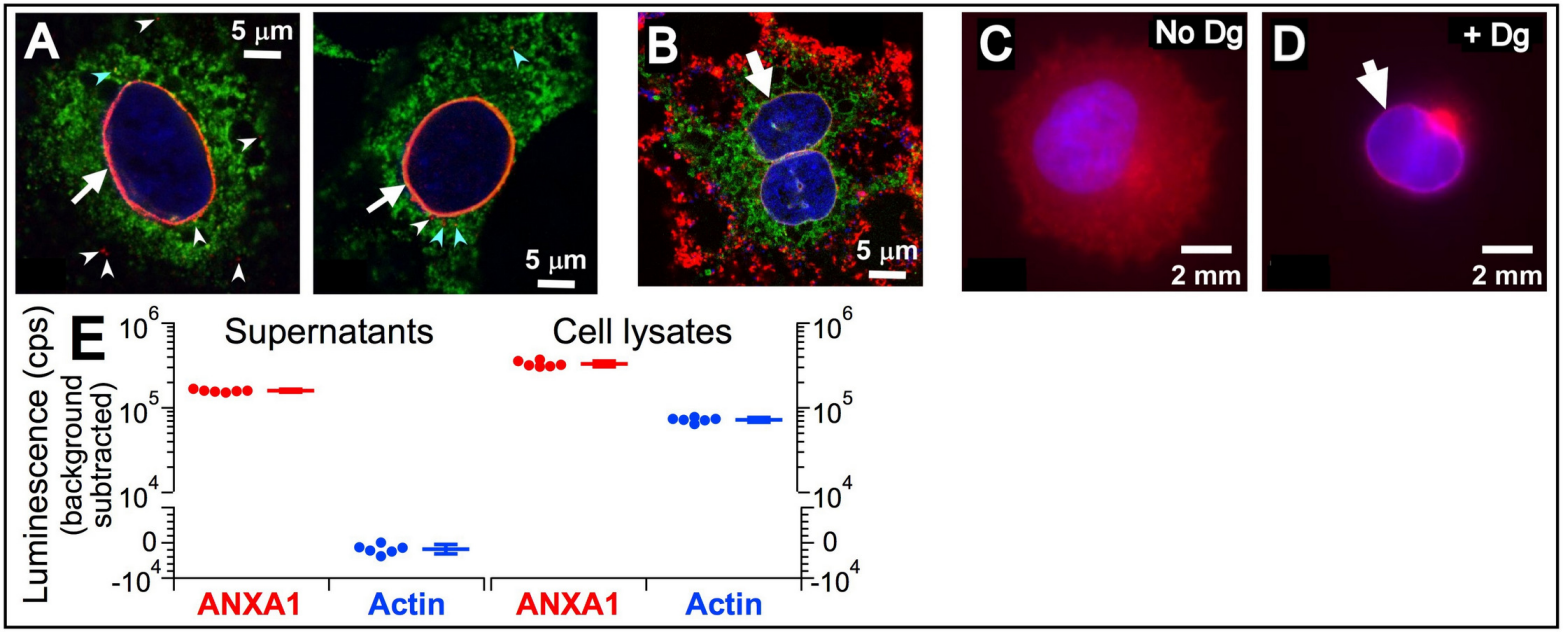
Localization of AnxA1 in the ER lumen. (**A***–***B**) Confocal images of permeablized A549 (**A**) and HeLa (**B**) cells. Native ANXA1 (red), calnexin (green), nuclei (blue). ANXA1 co-localized with calnexin in nuclear envelope (white arrow). ANXA1 also found in puncta without (white arrowhead) or with (cyan arrowhead) calnexin. (**C***–***D**) Confocal images of intact (**C**) and digitonin permeabilized (**D**) Panc-1 cells. Native ANXA1 (red), nuclei (blue). White arrow in (**D**) indicates nuclear envelope labeled with ANXA1. (**E**) ANXA1-(red) and β-actin-(blue) capture ELISA of supernatant (left) and whole-cell lysate (right). Dots: signals detected in individual assays (*n* = 6); horizontal bars: mean, s.e.m. Figure 6—source data 1.Abundance of annexin proteins (quantified as total spectral counts by mass spectrometry analysis) detected in experiment eluates collected from magnetic beads covalently linked to peptides with modified pL2 sequence, and in control eluates collected from beads linked to peptide with scrambled modified pL2 sequence.The spectral count entry is black when the count ratio (experiment count/control count) is ≥1.2; grey when 1.2 > count ratio≥1.0; and grey italics when count ratio <1.0. *lfsr* is the local false sign rate (see *Statistical analysis of mass spectrometry output* in the Materials and methods section). The *lfsr* ≤0.2 (corresponds to a global false discovery rate ≤5%) are bolded.

Among annexins, ANXA1 is most widely reported to be present in locations topologically equivalent to the ER lumen. To confirm that ANXA1 was present in the ER lumen, we localized it by immunofluorescence microscopy. A549 cells were briefly exposed to a low concentration of digitonin to permeabilize the PM to release cytoplasmic ANXA1. Remaining ANXA1 co-localized with the ER marker calnexin in the nuclear envelope and in cytoplasmic puncta (Figure 6A). ANXA1 localization to the nuclear envelope was also observed in HeLa and Panc-1 cells (Figure 6B–D). The inability to observe ANXA1 throughout the ER could be the result of its low expression in the bulk ER lumen. Therefore, to further establish localization to the ER lumen, we performed capture ELISA of secreted proteins. The non-secreted β-actin was detected only in the lysate (Figure 6E, blue), indicating that the HEKtsA201 cells remained healthy and intact during the 3 day incubation period. In contrast, the signal for extracellular ANXA1 was robust (Figure 6E, red), suggesting that ANXA1 was present in the ER lumen en route to be secreted.

**Figure 6—figure supplement 1.**
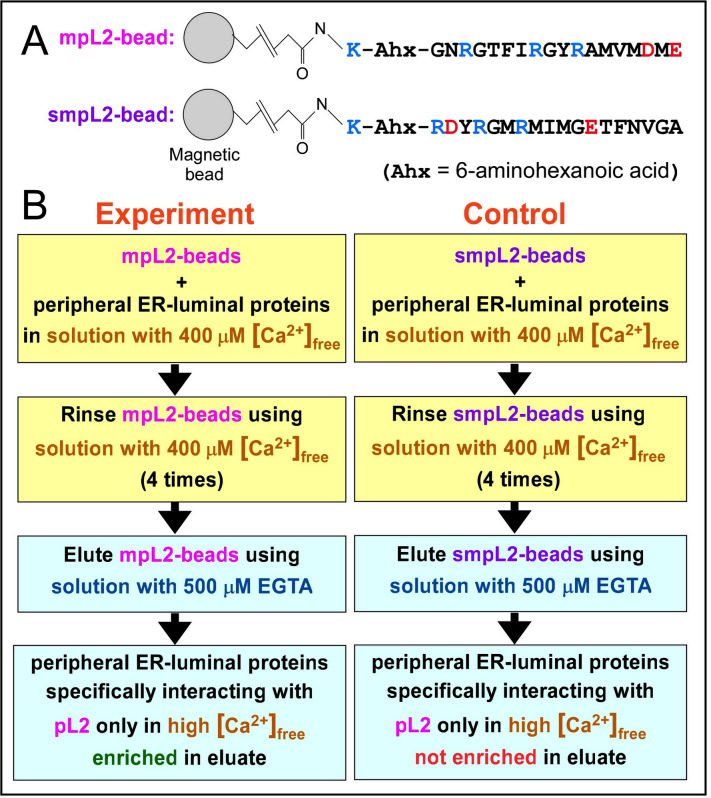
Pull-down of peripheral luminal proteins that interact with IP_3_R-3 L2 loop. (**A**) Peptides with modified pL2 (mpL2) or scrambled modified pL2 (smpL2) sequences were immobilized on magnetic beads. (**B**) Protocol to enrich soluble proteins from bovine ER-microsome fractions that interact with pL2 peptide in presence of high physiological [Ca^2+^].

### Luminal ANXA1 can mediate [Ca^2+^]_ER_-dependent InsP_3_R channel inhibition

**Figure 7.**
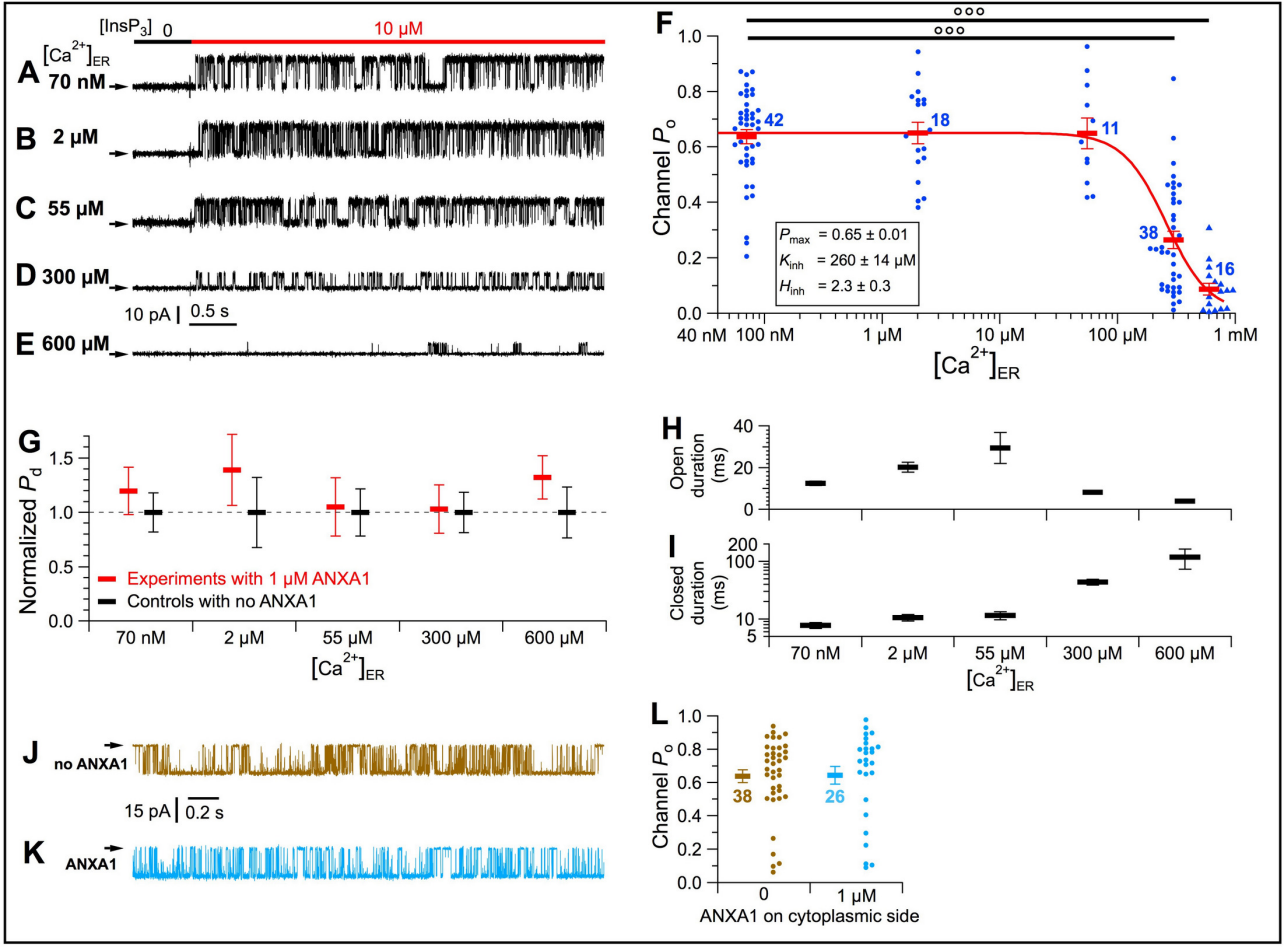
[Ca^2+^]_ER_ inhibition of InsP_3_R-3 channel activity is mediated by a specific interaction between the channel and ANXA1 in the ER lumen. (**A***–***E**) Typical current traces of InsP_3_R-3 channels in cyto-out configuration with 1 μM ANXA1 and various [Ca^2+^]_ER_ in pipette solution. In *C–E* with [Ca^2+^]_ER_ >40 μM, channel conductance reduced due to permeant-ion block. (**F**) *P*_o_ of individual current traces (blue symbols) and averages and s.e.m. (red horizontal bars) observed under conditions in (*A–E*). Red curve: least-squares fit to average *P*_o_ at various [Ca^2+^]_ER_ using simple inhibitory Hill equation with parameters tabulated. (**G**) Averages and s.e.m. of normalized probability of detection of InsP_3_R channels (*P*_d_) in cyto-out experiments with 0 (black) or 1 μM (red) ANXA1 in pipette solutions with various [Ca^2+^]_ER_. (**H***–***I**) Averages and s.e.m. of InsP_3_R-3 channel open (**H**) and closed (**I**) durations in cyto-out patch-clamp experiments in (**F**). (**J***–***K**) Typical current traces in on-nuc patches with pipette (cytoplasmic) solutions containing 2 μM Ca^2+^_i_ and 10 μM InsP_3_, with 0 (**J**) or 1 μM (**K**) ANXA1. Bath solution: 70 nM Ca^2+^_ER_ with 0-MgATP. (**L**) Po of individual current traces (brown: no ANXA1; light blue: 1 μM ANXA1 on cytoplasmic side) and averages and s.e.m. (respective horizontal bars) observed under conditions in (**J–K**).

We examined direct effects of ANXA1 on channel gating in cyto-out patch-clamp experiments with pipette solutions (bathing the luminal side) containing 1 μM recombinant full-length human ANXA1 under conditions that prevented permeant Ca^2+^ feed-through effects (Figure 7—figure supplement 1). ANXA1 was without effect on maximally-stimulated channel *P*_o_ (≈ 0.65) in [Ca^2+^]_ER_ between 70 nM and 55 μM (Figure 7A–C and F). In contrast, with [Ca^2+^]_ER_ >55 μM, channel *P*_o_ dropped abruptly with increasing [Ca^2+^]_ER_ (Figure 7D–F). Fitting the *P*_o_ data with a simple inhibitory Hill equation (Figure 7F):

(1) $$P_{o}=P_{max}{\left\{1+{\left(\frac{[Ca^{2+}{]}_{ER}}{K_{inh}}\right)}^{H_{inh}}\right\}}^{-1}$$

**Figure 7—figure supplement 1.**
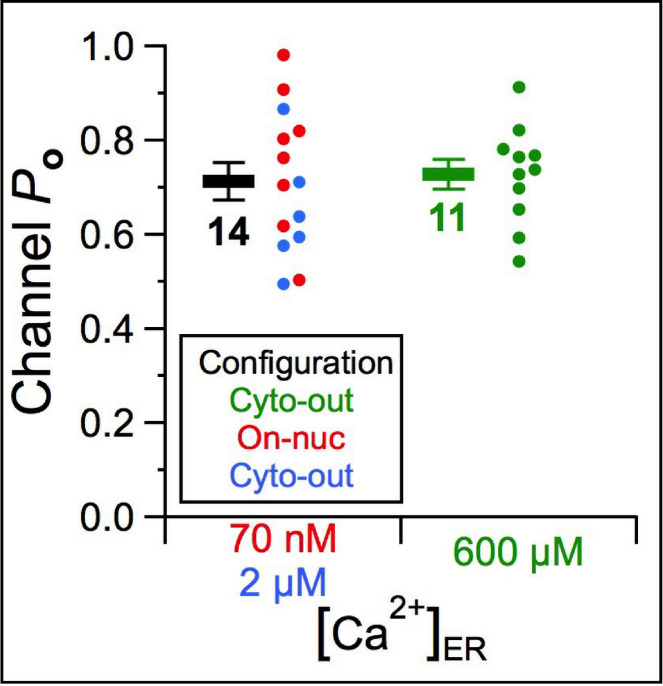
Abrogation of Ca^2+^ feed-through effect on InsP_3_R channel activity by buffering [Ca^2+^]_i_ with a high concentration (10 mM) of the Ca^2+^ chelator HEDTA in the cytoplasmic solution. Channel *P*_o_ of individual current traces (circles) and their averages and s.e.m. (horizontal bars) are plotted for cyto-out (green and blue circles) and on-nuc (red circles) patch-clamp experiments performed. There is no significant difference in channel *P*_o_ observed in the presence (green data) and absence (data in other colors) of Ca^2+^ flux driven through the active channel by high [Ca^2+^]_ER_. Channels were activated by perfusion solutions containing optimal 2 μM [Ca^2+^]_i_, saturating 10 μM [InsP_3_] and 0.5 mM [ATP^4-^].

showed that the half-maximal inhibitory [Ca^2+^]_ER_ (*K*_inh_) is 260 ± 14 μM with Hill coefficient (*H*_inh_) of 2.3 ± 0.3 (Figure 7F). The observed *K*_inh_ is within observed physiological levels of [Ca^2+^]_ER_ (~100 μM to 1 mM) (Zampese and Pizzo, 2012) and *K*_d_ of ANX binding to Ca^2+^. Without affecting the number of channels observed (Figure 7G), ANXA1 inhibited *P*_o_ mainly by increasing channel-closed durations (Figure 7H–I).

ANXA1 is primarily localized to the cytoplasm. However, ANXA1 on the cytoplasmic side of the InsP_3_R was without affect (Figure 7J–L). Thus, ANXA1 inhibits InsP_3_R activity exclusively from the luminal side.

### ANXA1 inhibits InsP_3_R channel gating by interacting with the L2 loop region

**Figure 8.**
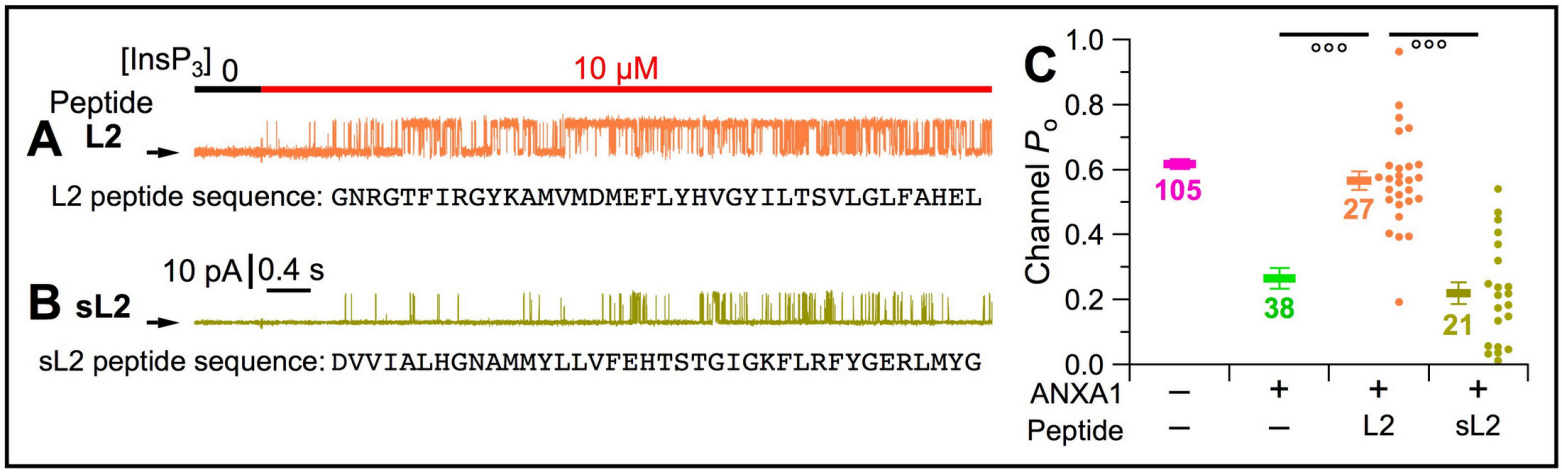
L2 peptide, but not scrambled L2 peptide, was able to compete ANXA1 from the InsP_3_R channel and restore high channel activity. (**A***–***B**) Typical current traces in cyto-out experiments with pipette solutions containing 300 Ca^2+^_ER_, 1 μM ANXA1, and 40 μM of L2 (**A**) or scrambled L2 (sL2) (**B**) peptides. Peptide sequences shown below corresponding traces. (**C**) *P*_o_ of individual current traces (orange: L2 peptide; yellow: sL2 peptide) and averages and s.e.m. (horizontal bars) observed under conditions in (**A–B**). For comparison, averages and s.e.m. of *P*_o_ in similar cyto-out experiments without peptide, and without (magenta bars) and with (light green bars) ANXA1, from Figures 4J and 6F, respectively.

To determine if ANXA1 inhibition is mediated by interaction with the L2 loop, we performed cyto-out patch-clamp experiments with the pipette solution facing the luminal aspect containing 1 μM ANXA1 and 40 μM of peptides with either the L2 sequence or a scrambled L2 (sL2) sequence (Figure 8A–B, respectively) to compete with the InsP_3_R for binding to ANXA1 (Figures 5J and 7F). The L2 peptide, but not the sL2 peptide, completely abrogated inhibition of InsP_3_R activity by ANXA1 (Figure 8C). This suggests that suppression of channel activity by ANXA1 is specifically mediated by the L2 region of the InsP_3_R.

### Native PLP-mediated and recombinant ANXA1-mediated [Ca^2+^]_ER_-dependent InsP_3_R channel inhibition have similar features

**Figure 9.**
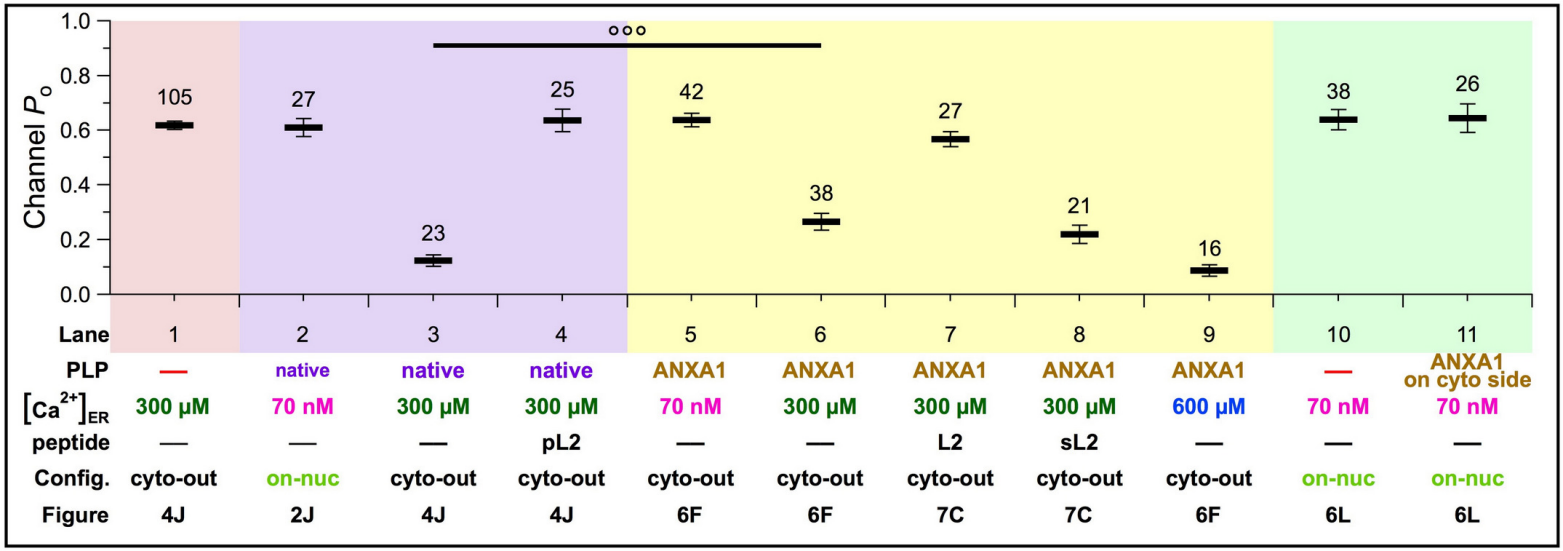
Modulation of homotetrameric InsP_3_R channel activity by native inhibitory peripheral luminal protein or ANXA1 under various experimental conditions: 70 nM, 300 or 600 μM Ca^2+^_ER_; in the absence or presence of peptides with L2 sequences (pL2, L2 or sL2); and in on-nuc or cyto-out patch-clamp configurations. Averages and s.e.m. of InsP_3_R-3 *P*_o_ are shown as tabulated.

In sub-physiological 70 nM Ca^2+^_ER_, neither the native PLP (Figure 9, Lane 2) nor recombinant ANXA1 (Figure 9, Lane 5) suppressed channel activities, since *P*_o_ observed were indistinguishable from those with no PLP on the luminal side (Figure 9, Lane 1). In 300 μM Ca^2+^_ER_, both native PLP (Figure 9, Lane 3) and luminal ANXA1 (Figure 9, Lane 6) inhibited the *P*_o_. Inhibition by PLP or ANXA1 was completely abrogated by L2 loop peptides (Figure 9, Lanes 4 and 7, respectively). Such similarities suggest that ANXA1 is the major PLP mediating [Ca^2+^]_ER_-dependent inhibition of InsP_3_R channels.

Although native PLP and luminal ANXA1 both inhibit InsP_3_R activity, *P*_o_ of PLP-inhibited InsP_3_R channels (Figure 9, Lane 3) was lower than that of ANXA1-inhibited channels (Figure 9, Lanes 6 and 8). However, in higher [Ca^2+^]_ER_ (600 μM), ANXA1-inhibited channel *P*_o_ (Figure 9, Lane 9) was comparable to the native PLP-inhibited *P*_o_ (Figure 9, Lane 3). This suggests that the luminal concentration of endogenous ANXA1 is effectively higher than 1 μM, and/or that other factor(s) enhance the [Ca^2+^]_ER_ sensitivity of the channel to inhibition by ANXA1.

### Annexin A1 specifically mediates [Ca^2+^]_ER_-dependent InsP_3_R channel inhibition

**Figure 10.**
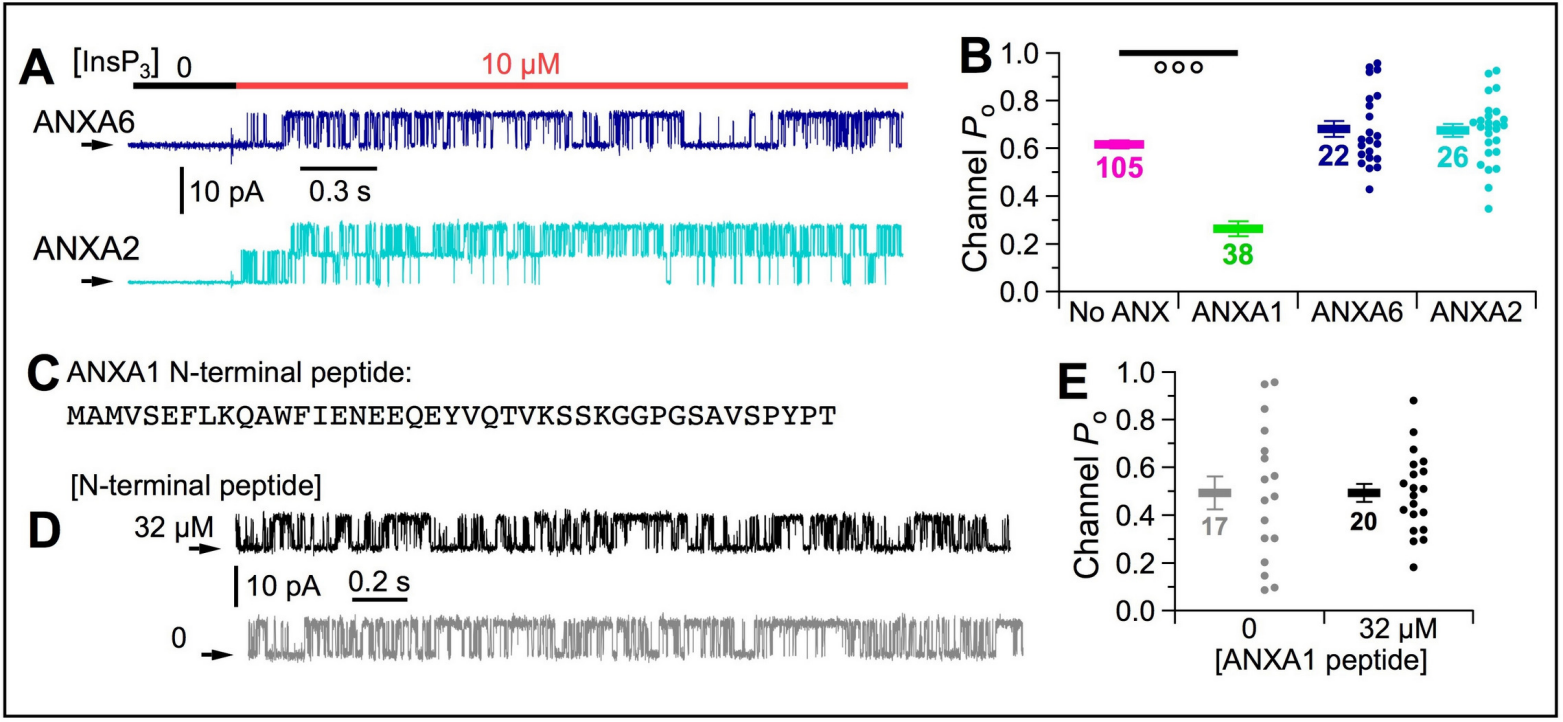
[Ca^2+^]_ER_ inhibition of InsP_3_R channel activity is specifically mediated by ANXA1 through interaction with the L2 loop of InsP_3_R. (**A**) Typical current traces of InsP_3_R-3 in cyto-out experiments with pipette containing 300 μM Ca^2+^_ER_ and 1 μM ANXA6 or A2. Isolated patches perfused with 2 μM Ca^2+^_free_, 0.5 mM ATP^4–^ with 0 or 10 μM InsP_3_ as indicated. (**B**) *P*_o_ of individual current traces (circles) and average and s.e.m. (horizontal bars) for cyto-out experiments with ANXA6 (blue) or ANXA2 (cyan). For comparison are averages and s.e.m. of *P*_o_ in similar experiments with no ANXA1 (magenta bars) and ANXA1 (green bars), from Figures 5J and 6F, respectively. (**C**) Synthetic peptide with 41 residues of N-terminal domain of ANXA1. (**D**) Typical current traces of InsP_3_R-3 in cyto-out configuration with pipette containing 300 μM Ca^2+^_ER_, with or without 32 μM ANXA1 N-terminal peptide. Isolated patches perfused with solution containing 2 μM Ca^2+^_i_, 10 μM InsP_3_ and 0.5 mM ATP^4–^. (**E**) *P*_o_ of individual current traces (circles) and average and s.e.m. (horizontal bars) for cyto-out experiments with (black) or without (grey) 32 μM ANXA1 N-terminal peptides.

Multiple annexins were identified in our mass spectroscopy analysis, including ANXA6, ANXA2 and ANXA11 (Figure 6—source data 1). However, recombinant ANXA6 or ANXA2 had no effect on channel *P*_o_ (Figure 10A–B). Similar experiments with ANXA11 were precluded by its low solubility. The ANXA6 used had an N-terminal His tag. However, suppression of InsP_3_R activities by ANXA1 with and without an N–terminal His tag was equivalent (Figure 10—figure supplement 1), indicating that the observed effects were not impacted by presence or absence of the tag.

**Figure 10—figure supplement 1.**
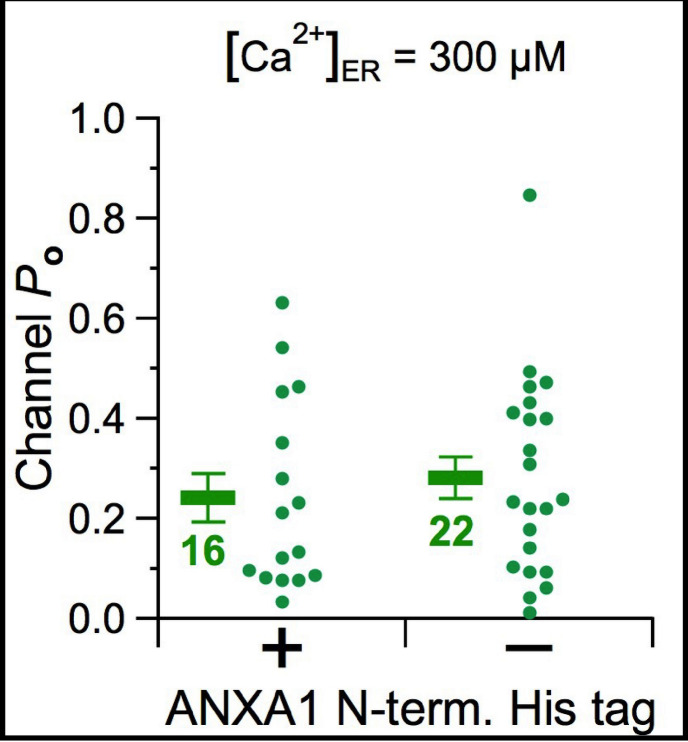
Presence of a His tag at the N-terminus of the ANXA1 has no effect on its ability to inhibit InsP_3_R channel activities in 300 μM [Ca^2+^]_ER_. InsP_3_R-3 channel *P*_o_ of individual current traces (green circles), the averages and s.e.m. of *P*_o_ (green horizontal bars) for cyto-out experiments performed using ANXA1 with or without His tag at its N-terminal are plotted as indicated. Channels in the cyto-out patch-clamp configuration were activated by perfusion solutions containing optimal 2 μM [Ca^2+^]_i_, saturating 10 μM [InsP_3_] and 0.5 mM [ATP^4-^].

Annexins have a conserved C-terminal ‘annexin core’ domain with multiple ‘annexin-type’ Ca^2+^-binding sites (Gerke and Moss, 2002). In contrast, the N-terminal regions are diverse (Raynal and Pollard, 1994). That ANXA1, but neither ANXA6 nor ANXA2, inhibited gating of InsP_3_R channels suggests that inhibition is mediated by interaction of its N-terminal domain with the InsP_3_R. To determine if the N-terminal domain itself can affect channel activity, we included 32 μM N-terminal peptide of human ANXA1 (Figure 10C) in the pipette solution with 300 μM Ca^2+^_ER_, and found that channel *P*_o_ was unaffected (Figure 10D–E). This suggests that whereas the N-terminal domain may mediate interaction between ANXA1 and the InsP_3_R L2 region, the ANXA1 C-terminal domain is likely needed to localize ANXA1 to the two-dimensional surface of the ER membrane to increase its local concentration to facilitate N-terminus binding to the InsP_3_R to suppress channel activity.

### Endogenous ANXA1 inhibits InsP_3_R-mediated Ca^2+ ^release in intact cells

**Figure 11.**
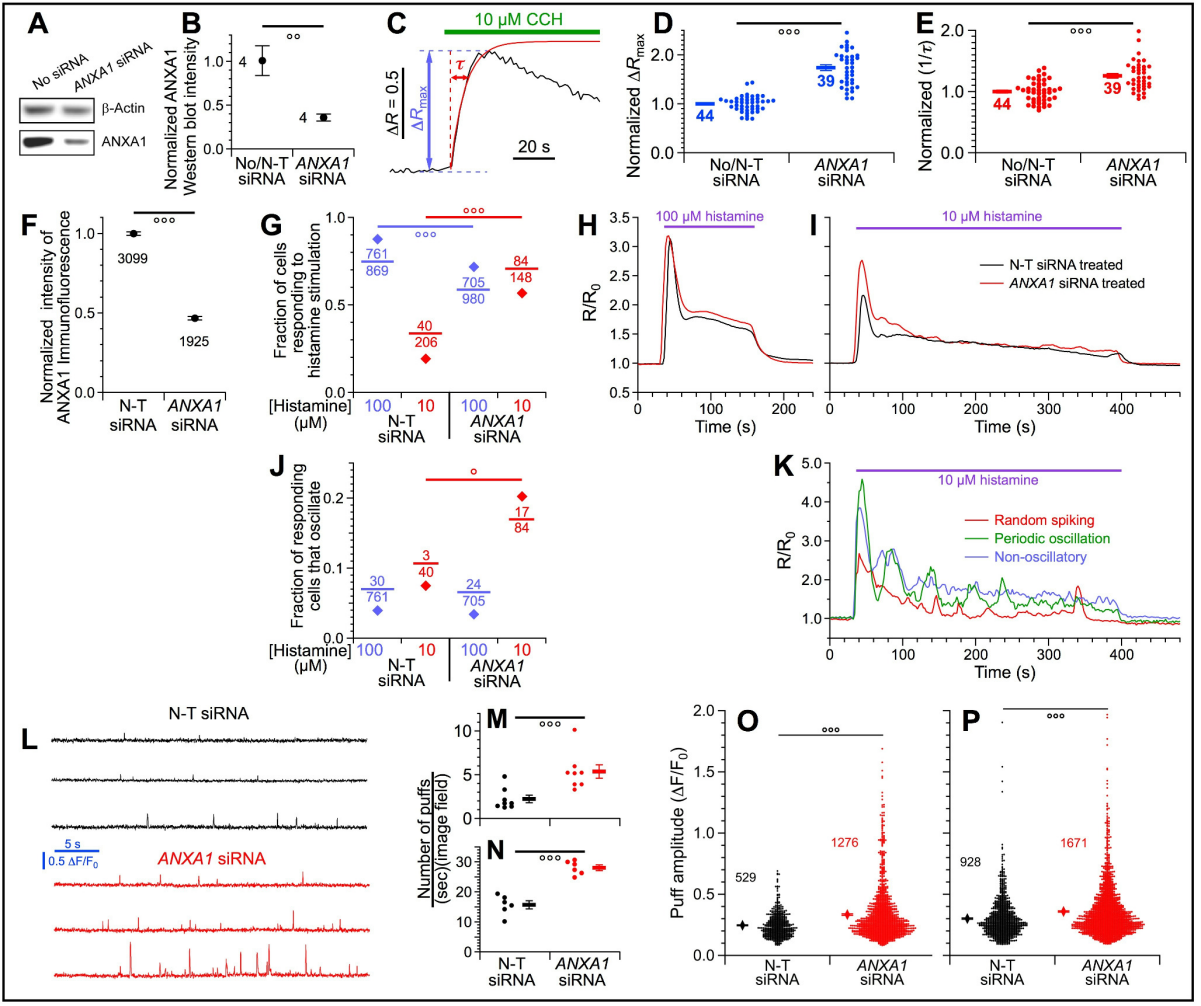
Endogenous ANXA1 inhibits InsP_3_R-mediated Ca^2+^ release. (**A**) Western blot of ANXA1 and β-actin in lysates from HEKtsA201 cells treated with transfection medium (left) or *ANXA1* siRNA (right) (one of four similar blots shown). (**B**) Summary of ANXA1 protein knockdown. HEKtsA201 cells treated with *ANXA1* siRNA (four samples), transfection medium only (two samples) or with non-targeting (N-T) siRNA (two samples). (**C**) Typical trace of fura-2 fluorescence ratio (Δ*R*) observed in population of intact HEKtsA201 cells stimulated by 10 μM carbachol (CCH). Maximal rise in Δ*R* (Δ*R*_max_): blue double arrow; time constant (*τ*) of single exponential fit to rising phase of fluorescence ratio (red curve) is time for Δ*R* to rise to [1–1/*e*] of Δ*R*_max_: red double arrow. (**D**) Normalized Δ*R*_max_ of individual Δ*R* traces (circles) and averages and s.e.m. (horizontal bars) for *ANXA1* siRNA-treated (right) and control cells (left). Number of traces tabulated next to horizontal bars. (**E**) Normalized rate of change of Δ*R* (1/*τ*) for Δ*R* traces using convention as (**D**). (**F**) ANXA1 immunofluorescence intensity of HeLa cells treated with non-targeting (N-T) or *ANXA1* siRNA. Number of cells tabulated below corresponding circles. (**G**) Fractions of N-T or *ANXA1* siRNA-treated HeLa cells that responded by ER Ca^2+^ release through InsP_3_R when stimulated by sub-saturating 10 (red) or saturating 100 (blue) μM histamine. (**H–I**) Traces of mean normalized ER Ca^2+^ release from N-T (red) or *ANXA1* (black) siRNA-treated HeLa cells responding to 100 μM (**H**) or 10 μM (**I**) histamine. (**J**) Fractions of N-T or *ANXA1* siRNA-treated HeLa cells that oscillated in response to 10 (red) or 100 (blue) μM histamine. (**K**) Selected traces showing different kinds of Ca^2+^ signals in *AnxA1* siRNA-treated HeLa cells responding to sub-saturating 10 μM histamine. (**L**) Typical fluorescence amplitude (ΔF/F_0_) traces showing local Ca^2+^ release events (puffs) in HEK293 cells treated with N-T (black) or *ANXA1* (red) siRNA. Cells stimulated by photolysis of caged i-InsP_3_ using sub-maximal 50 ms UV flash. (**M–P**) Puffs generated by 50 ms UV flash were subsequently observed for 30 s in eight imaging fields (**M**); puffs generated by maximal 150 ms UV flash and subsequently observed for 10 s in six imaging fields (**N**). Dots indicate numbers of puffs observed for N-T (black) and *ANXA1* (red) siRNA-treated cells. Means and s.e.m. indicated by bars. (**O–P**) Dot plots of ΔF/F_0_ of individual Ca^2+^ puffs observed in N-T (black) and *ANXA1* (red) siRNA-treated cells, generated by 50 ms (**O**) and 150 ms (**P**) UV flashes, respectively. Means and s.e.m. indicated by diamonds and bars, respectively.

To determine effects of ANXA1 on cytoplasmic Ca^2+^ signaling, we used siRNA to reduce ANXA1 protein expression after 48 hr to ~35% of WT levels in HEKtsA201 cells (Figure 11A–B), and measured magnitude (Δ*R*_max_) and rate of change (1/*τ*) of Fura-2 fluorescence ratio in populations of cells responding to the muscarinic receptor agonist carbachol (Figure 11C) in a bath solution containing 1.5 mM CaCl_2_. These parameters capture the initial InsP_3_R-mediated Ca^2+^-release phase of the response to agonist. Both parameters were enhanced specifically in the *ANXA1* siRNA-treated cells (Figure 11D–E, respectively), suggesting that endogenous ANXA1 inhibits InsP_3_R-mediated Ca^2+^ release.

We also measured histamine-induced changes in Fura-2 fluorescence ratio (R/R_0_) in individual *ANXA1* and non-targeting (N-T) siRNA-treated HeLa cells (Figure 11F). Saturating (100 µM) histamine elicited similar responses in both kinds of siRNA-treated cells (Figure 11H). In contrast, in sub-saturating (10 µM) histamine, an increase in both the fraction of responding cells (Figure 11G) and the maximal increase in R/R_0_ was observed in the *ANXA1* siRNA-treated cells (Figure 11I). We classified responding cells as ‘oscillatory’ or ‘non-oscillatory’ (Figure 11K). In 100 µM histamine, the fraction of ocillatory cells was indistinguishble between *AnxA1* or N-T siRNA-treated cells; whereas in 10 µM histamine, a significantly higher fraction of *ANXA1* siRNA-treated cells showing either ‘random spiking’ or ‘periodic oscillations’ (Figure 11J).

We explored this further by visualizing elementary Ca^2+^-release events (Ca^2+^ puffs) generated by clusters of InsP_3_R stimulated by uncaging of a non-metabolizable InsP_3_ analogue (Figure 11L; Lock et al., 2018). More Ca^2+^ puffs were observed in the cells treated with *ANXA1*-siRNA than in cells treated with non-targeting siRNAs (Figure 11M–N), with larger amplitudes (Figure 11O–P), regardless of whether they were generated by a sub-saturating UV flash (50 ms, Figure 11M and O) or a saturating one (150 ms, Figure 11N and P). Taken together, these results suggest that endogenous luminal ANXA1 inhibits InsP_3_R-mediated Ca^2+^ release in vivo, in strong agreement with the strong inhibition by ANXA1 of InsP_3_R channel activity observed in vitro.

## Discussion

### A novel mechanism of strong regulation of InsP_3_R channel activity by ER [Ca^2+^]

Our study has established that physiological [Ca^2+^]_ER_ in the normally-replete ER lumen strongly inhibits the activity of the InsP_3_R Ca^2+^-release channel, with consequent effects on InsP_3_-mediated Ca^2+^ signals in cells. This inhibitory effect is not mediated by direct Ca^2+^ binding to luminal sites on the InsP_3_R nor by flux-through effects of Ca^2+^ on cytoplasmic regulatory sites. Rather, it is mediated by a direct interaction of the InsP_3_R with a lumen-localized membrane-associated protein, which is likely to be ANXA1.

We first established that high [Ca^2+^]_ER_ strongly inhibits InsP_3_R channel activity by a mechanism involving a putative ER-luminal peripheral protein. To identify the protein, we focused on a segment that is highly conserved in all three InsP_3_R isoforms and which, according to a 2015 cryo-EM structure (Fan et al., 2015), is located as a luminal loop (L2) between transmembrane helices TM3 and TM4 of InsP_3_R-1. Interestingly, the L2 sequence was assigned to a different location in a recent cryo-EM structure of InsP_3_R-3 (Paknejad and Hite, 2018). Nevertheless, we discovered ANXA1 by pull-down with the pL2 peptide, and inhibition of channel activity by high [Ca^2+^]_ER_ was relieved by inclusion of L2 peptides in the ER lumen. These experiments establish that the inhibition of InsP_3_R activity is mediated through a protein interaction with the InsP_3_R L2 region. The structures in Paknejad and Hite, 2018 have closed channel pores despite binding of InsP_3_ and Ca^2+^. Of note, ANXA1 reduces, but does not completely abrogate activity of the InsP_3_R. It is possible that the topology of the L2 region is different in functionally distinct channel-activation states, with some states enabling it to interact in the ER lumen with ANXA1.

Whereas the predominance of our data suggests that the luminal protein responsible for InsP_3_R inhibition in high [Ca^2+^]_ER_ is ANXA1, some results give us pause. First, we have been unable to co-immunoprecipitate (co-IP) the two proteins from cell lysates. There are many reasons why co-IP can fail, including low expression of ANXA1 in the ER lumen, particularly in comparison with its strong expression in the cytoplasm; and that the interacting regions in InsP_3_R and ANXA1 are small, particularly in the case of the InsP_3_R. Second, whereas we have been able to apply immunofluorescence microscopy to localize ANXA1 to the nuclear envelope in different permeabilized cells with cytoplasmic ANXA1 washed out, localization to the ER has been unconvincing (Figure 3A–E). This may reflect a very low abundance of luminal ANXA1 in the peripheral ER that escapes detection. Third, effects of ANX1A knockdown on intracellular Ca^2+^ signaling were modest (Figures 11D–E,G–J,M–P). This could be due to the fact that knockdown reduced the ANXA1 expression level by only about 65% (Figure 11B and F). Taken together, these results may suggest that ANXA1 is not the *bona fide* PLP. Other ER-luminal proteins have also been reported to regulate InsP_3_R activity, but their effects are all very different from those of the PLP defined here. Direct interaction with chromogranins enhances activity (Yoo and Lewis, 2000), and BiP/Grp78 promotes tetramerization of InsP_3_R-1, thereby enhancing Ca^2+^ release (Higo et al., 2010). In contrast, the PLP here inhibits InsP_3_R channel activity. Thioredoxin ERp44 interacts directly with InsP_3_R to suppress channel activity (Higo et al., 2005). However, ERp44-mediated inhibition is reduced as [Ca^2+^]_ER_ increases beyond 100 μM, a [Ca^2+^]_ER_ dependence opposite to that of the PLP defined here. Furthermore, ERp44 interacts specifically with InsP_3_R-1, whereas the PLP here inhibits all InsP_3_R isoforms.

Annexins are defined by a conserved C-terminal Ca^2+^ binding domain (Gerke and Moss, 2002). Although the intrinsic affinity of ‘annexin-type’ Ca^2+^ binding sites is low (*K*_d_ up to mM range) (Raynal and Pollard, 1994), the presence of phospholipids, especially those with negatively-charged headgroups, strongly enhances Ca^2+^ affinity (Gerke and Moss, 2002). Conversely, the affinity of annexins for acidic phospholipids increases dramatically in the presence of high [Ca^2+^]_free _(Raynal and Pollard, 1994; Gerke and Moss, 2002). This feature is likely the mechanism underlying the [Ca^2+^]_ER_ dependence of ANXA1 inhibition of InsP_3_R activity. In low [Ca^2+^]_ER_, ANXA1, having low affinity for phospholipids, remains in the bulk ER lumen, rendering an interaction between ANXA1 and InsP_3_R channels that are confined to the ER membrane highly unfavorable entropically. With no ANXA1 interaction, the channel can gate robustly. In contrast, in high [Ca^2+^]_ER_ (>100 μM, Figure 7F), the ANXA1 C-terminus binds to phospholipids with high affinity and become restricted to the surface of the ER membrane. This substantially increases the chances for ANXA1 to interact with the InsP_3_R, which promotes channel inhibition.

### Physiological implications of ANXA1 regulation of the InsP_3_R

Inhibition of InsP_3_R by the PLP is very potent. With [Ca^2+^]_ER_ sufficiently high (≥300 μM), the PLP and ANXA1 strongly suppressed channel activation even in optimal concentrations of cytoplasmic Ca^2+^ and InsP_3_, such that channel *P*_o_ is limited to ~0.1 (Figure 7F), merely 15% of the maximal *P*_o_ elicited by saturating [InsP_3_] in absence of PLP (ANXA1) (Vais et al., 2012). This powerful effect is comparable in magnitude to the maximal inhibition of InsP_3_R activity by high (50 μM) [Ca^2+^]_i _(Vais et al., 2012). Furthermore, the range of [Ca^2+^]_ER_ over which this regulation occurs is within the physiological range. [Ca^2+^]_ER_ in fully-replete stores has been measured to be 250 μM – 1 mM (Zampese and Pizzo, 2012). We observed strong channel inhibition at 300 μM, with more profound inhibition at 600 μM. During agonist stimulation, bulk [Ca^2+^]_ER_ falls to concentrations that activate STIM1, which has an apparent luminal-Ca^2+^ affinity of 200–400 μM (Suzuki et al., 2014; Stathopulos et al., 2008). We observed apparent Ca^2+^ affinity of ANXA1-mediated inhibition of ~250 μM, suggesting that relief of inhibition will occur under physiological conditions that activate STIM1 and store-operated Ca^2+^ entry.

It has been suggested that most InsP_3_R channels (~95%) in cells are unresponsive to experimentally-induced elevations of [InsP_3_], with responsive channels limited to those in immobile clusters from which Ca^2+^ blips and puffs originate (Lock et al., 2019; Keebler and Taylor, 2017; Thillaiappan et al., 2017). The mechanisms that silence the majority of InsP_3_R are unknown, but our results indicate that the PLP defined here (ANXA1) could play a role. In this scenario, most InsP_3_R are normally associated with the PLP, rendering them unresponsive to InsP_3_. However, channels in some clusters may lose their association with the PLP, possibly because of a unique phospholipid composition of the ER membrane there, or because the L2 sequence is not exposed in InsP_3_R channels in clusters, or for other reasons. Channels in such clusters would be much more responsive to InsP_3_ and ‘licensed’ to respond even with the ER fully replete with Ca^2+^. In addition, the strong inhibition of InsP_3_R channel activity by ANXA1 could possibly help shape intracellular InsP_3_-induced Ca^2+^ signals. PLP (ANXA1) inhibition will reduce the amount of Ca^2+^ released by shortening the open durations of activated channels (Figure 7H) and reducing their frequency by lengthening their closed durations (Figure 7I). With fewer Ca^2+^ release events with reduced magnitude, coordination by released Ca^2+^ from channels in a cluster to generate Ca^2+^ puffs can be hampered, preventing puffs from being organized into global Ca^2+^ signals. Such suppression of Ca^2+^ release by the PLP could ensure spatial fidelity of local Ca^2+^ signals and be a fail-safe mechanism to prevent excessive, detrimental release of Ca^2+^ from the ER. At reduced [Ca^2+^]_ER_, the PLP regulation of InsP_3_R activity could also be a mechanism to preserve fidelity of Ca^2+^ signals. As [Ca^2+^]_ER_ drops, the [Ca^2+^] gradient across the InsP_3_R is reduced, and the Ca^2+^ flux it drives through the pore decreases. Between 100 and 600 μM [Ca^2+^]_ER_, the six-fold reduction in Ca^2+^ flux could be compensated by the ~6.6 fold increase in the *P*_o_ of the InsP_3_R channels (from ~0.09 to 0.6) as PLP inhibition of channel activity is alleviated (Figure 7F). This could allow the amount of Ca^2+^ released to be maintained despite the drop in [Ca^2+^]_ER_ to preserve Ca^2+^ signals required to regulate downstream cellular processes.

### Limitations of the current study

1. As noted, co-immunoprecipitation of ANXA1 with the InsP_3_R was not successful. Further studies, using different methods and antibodies should be performed to determine whether the proteins physically interact.
2. As noted, ANXA1 was secreted into the bath (Figure 6E), but its localization in permeabilized cells was evident only in the nuclear envelope (Figure 6A–D), and not in the peripheral ER. On the other hand, AnxA1 was initially identified as a potential protein that interacts with the InsP_3_R through in-vitro pull-down assays and mass spectrometry, using ultra-centrifuged ruptured ER microsome fraction devoid of nuclei. Additional studies employing different antibodies and imaging techniques, including super-resolution light microscopy and electron microscopy should be performed to establish the presence of ANXA1 in the lumen of the entire ER.
3. We demonstrated increased channel *P*_o_ in the presence of the pL2 peptide and concluded that it was caused by the peptide reversal of the inhibition by luminal Ca^2+^. A control experiment to demonstrate that the pL2 peptide does not increase channel *P*_o_ under conditions with reduced luminal [Ca^2+^] should be performed. We note however that the channel *P*_o_ is already very high under conditions of reduced luminal [Ca^2+^]. In a control experiment, we could change the conditions, for example use lower [InsP_3_] to observe channels with a low channel *P*_o_ in conditions of reduced luminal [Ca^2+^].

## Materials and methods

**Key resources table:** 

Reagent type (species) or resource	Designation	Source or reference	Identifiers	Additional information
Peptide, recombinant protein	Anx A1	Abcam	ab86446	
Peptide, recombinant protein	Anx A1 with N-terminal His tag	Abcam	ab184588	
Peptide, recombinant protein	Anx A2	Abcam	ab93005	
Peptide, recombinant protein	Anx A6	Abcam	ab92934	
Peptide, recombinant protein	L2 peptide (GNRGTFIRGYKAMVMDMEFLYHVGYILTSVLGLFAHEL)	Peptide 2.0	custom	
Peptide, recombinant protein	sL2 peptide (DVVIALHGNAMMYLLVFEHTSTGIGKFLRFYGERLMYG)	Peptide 2.0	custom	
Peptide, recombinant protein	pL2 peptide (GNRGTFIRGYKAMVMDME)	Peptide 2.0	custom	
peptide, recombinant protein	mpL2 peptide (K-Ahx-GNRGTFIRGYRAMVMDME, Ahx stands for 6-aminohexanoate residue)	Peptide 2.0	custom	
Peptide, recombinant protein	smpL2 peptide (K-Ahx-RDYRGMRMIMGETFNVGA, Ahx stands for 6-aminohexanoate residue)	Peptide 2.0	custom	
Peptide, recombinant protein	ANXA1 N-terminal peptide (MAMVSEFLKQAWFIENEEQEYVQTVKSSKGGPGSAVSPYPT)	Peptide 2.0	custom	
Chemical compound, drug	siRNA buffer	Dharmacon	B-002000-UB-100	
Chemical compound, drug	siRNA transfection reagent	Dharmacon	T-2001–02	
Chemical compound, drug	diBrBAPTA (5,5′-dibromo1,2-bis(o-amino phenoxy)ehane -N,N,N′,N′-tetraacetic acid)	Invitrogen	D-1211	
Chemical compound, drug	diBrBAPTA (5,5′-dibromo1,2-bis(o-amino phenoxy)ehane -N,N,N′,N′-tetraacetic acid)	Santa Cruz Biotechnology	sc-2273516	
Chemical compound, drug	2Hydroxyethyl)ethylenediaminetriacetic acid (HEDTA)	Sigma	H7154	
Chemical compound, drug	inositol 1,4,5-trisphosphate	Invitrogen	I-3716	
Chemical compound, drug	inositol 1,4,5-trisphosphate	Santa Cruz Biotechnology	sc-201521	
Chemical compound, drug	NHS-activated magnetic beads	Pierce	88826	
Chemical compound, drug	Protein A Dynabeads	ThermoFisher	10006D	
Chemical compound, drug	Anti-Flag M2 agarose beads	Sigma	A2220	
Chemical compound, drug	Fura-2 AM	Molecular Probes	I-1225	
Chemical compound, drug	siGLO Red transfection indicator	Dharmacon	D-001630-02-05	
Chemical compound, drug	Cal-520/AM	AAT Bioquest	21130	
Chemical compound, drug	Caged Ins(1,4,5)P3/PM (caged InsP3)	Sirius Fine Chemical SiChem GmbH	cag-iso-2–145-	
Chemical compound, drug	EGTA/AM	ThemoFisher	E1219	
Cell line (Gallus gallus)	DT40 cells (wild-type)	Riken Bioresource Center	RCB1464; RRID:CVCL_0249	
Cell line (Gallus gallus)	DT40-KO cells (with all three InsP_3_R genes disrupted)	Riken Bioresource Center	RCB1467; RRID:CVCL_4634	
Cell line (Gallus gallus)	DT40-r3 cells	ref. (Mak et al., 2013b) in this study	NA	
Cell line (Homo-sapiens)	HEK293 cells	ATCC	CRL-1573; RRID:CVCL_0045	
Cell line (Homo-sapiens)	HEK-3KO cells	Kerafast	EUR030; RRID:CVCL_HB82	
Cell line (Homo-sapiens)	HEK293-3KO-r InsP3R-3 cells	this study	NA	
Cell line *Mus musculus*	N2a cells	ATCC	CCL-131; RRID:CVCL_0470	
Cell line (Rattus rattus)	PC12 cells	ATCC	CRL-1721; RRID:CVCL_0481	
Cell line (Homo-sapiens)	tsA201 cells	Sigma-Aldrich	96121229; RRID:CVCL_2737	
Cell line (Homo-sapiens)	A549 cells	ATCC	CCL-185; RRID:CVCL_0023	
Genetic reagent (*Homo sapiens*)	Anx A1 siRNA	Dharmacon	M-011161-01-0005	
Genetic reagent (*Homo sapiens*)	Non-targeting siRNA	Dharmacon	D-001206-13-05	
Antibody	rabbit polyclonal anti-AnxA1 antibody	Proteintech	21990–1-AP; RRID:AB_11182596	WB: 1:1000-1:4000 IP: 1:1000-1:10000 IHC: 1:50-1:500 IF: 1:20-1:200
Antibody	mouse monoclonal anti-AnxA1 antibody	ECM Biosciences	AM0211	ELISA 1:1000 ICC 1:100 IP 1:100 WB 1:1000
Antibody	rabbit polyclonal anti-FLAG antibody	Cell Signaling	14793S; RRID:AB_2572291	WB: 1:1000 IP: 1:50 IHC: 1:800 IF: 1:800 FC: 1:1600 Chromatin IP: 1:50
Antibody	goat anti-rabbit IgG (H+L) Cross-Adsorbed Secondary Antibody, Alexa Fluor 568	Invitrogen	A-11011; RRID:AB_143157	FC: 1–10 µg/mL ICC: 2 µg/mL IF: 2 µg/mL
Antibody	mouse monoclonal anti-calnexin antibody	Chemicon	MAB3126 RRID:AB_143157	ICC: 1:100-1:250 WB: 1:200-1:2000 IP: 1:200-1:1000
Antibody	rabbit polyclonal anti-β actin antibody	Cell Signaling	7881S; RRID:AB_1549731	capture Elisa: 1:100
Antibody	goat polyclonal anti-mouse IgG-HRP antibody	Cell Signaling	7074S; RRID:AB_2099233	capture Elisa: 1:1000-1:3000
Antibody	horse polyclonal anti-mouse IgG-HRP antibldy	Cell Signaling	7076S; RRID:AB_330924	capture Elisa: 1:1000-1:3000
Antibody	mouse monoclonal anti-βactin antibody	Cell Signaling	8H10D10; RRID:AB_2242334	WB: 1:1000 IHC: 1:8000-1:32000 IF: 1:2500-1:10000 FC: 1:200-1:800
Antibody	mouse monoclonal anti-type 3 InsP3R antibody	BD Transduction Laboratories	610312; RRID:AB_397704	WB: 1:2000-1:4000
Software, algorithm	QuB	refs. (Qin et al., 2000) and (Bruno et al., 2013) in this study		Quantitative single-channel analysis
Software, algorithm	IGOR Pro	Wavemetrics		Figure production and data fitting
Software, algorithm	Metamorph v7.7	Universal Imaging/Molecular Devices		Image analysis
Software, algorithm	Flika	Ellefsen et al., 2014		Image processing
Software, algorithm	Microcal Origin v6.0	OriginLab		Data analysis and graphing
Software, algorithm	Max Chelator	online freeware		Calculation of ion concentrations
Software, algorithm	MaxQuant, version 1.6.1.0	online freeware		Database search

### Proteins

Recombinant full-length human annexin proteins from Abcam were used in our experiments: ANXA1 (cat. # ab86446 with no tag; ab184588 with N-terminal His tag), Anx A2 (cat. # ab93005 with N-terminal His tag), and Anx A6 (cat. # ab92934 with N-terminal His tag).

### Synthetic peptides

Peptides used in our study: L2 (GNRGTFIRGYKAMVMDMEFLYHVGYILTSVLGLFAHEL), sL2 (DVVIALHGNAMMYLLVFEHTSTGIGKFLRFYGERLMYG), pL2 (GNRGTFIRGYKAMVMDME), mpL2 (K-Ahx-GNRGTFIRGYRAMVMDME, Ahx stands for 6-aminohexanoate residue), smpL2 (K-Ahx-RDYRGMRMIMGETFNVGA), and ANXA1 N-terminal peptide (MAMVSEFLKQAWFIENEEQEYVQTVKSSKGGPGSAVSPYPT) were custom synthesized by Peptide 2.0 (Chantilly, VA).

### Generation and maintenance of cell lines

All cell lines used were obtained from commercial sources and were routinely tested and confirmed to be mycoplasma-free via PCR. Wild-type DT40 chicken B cells and DT40-KO cells in which all three endogenous InsP_3_R genes have been stably ablated were obtained from Riken Bioresource Center, Japan (cell bank # RCB1464 and RCB1467, respectively). Generation of DT40–KO-r–InsP_3_R–3 cells (DT40-r3 cells) that stably express only recombinant rat type 3 InsP_3_R in DT40-KO cells was described in Mak et al., 2005. Wild type DT40 and DT40-r3 cells used in this study were grown in suspension culture in RPMI 1640 medium with 2 mM L-glutamine (Gibco cat. # 11875–085), supplemented with 10% (v/v) FBS, 1% chicken serum, and 1% Gibco antibiotic-antimycotic, and 1% G418 for selection; at 37°C in 5% CO_2_. When cell density exceeded 2.5 × 10^6^/ml, they were sub-cultured to 0.1–0.2 × 10^6^/ml (Mak et al., 2005).

Wild type HEK293 cells from ATCC (CRL-1573) and InsP_3_R-Null HEK-293 cells with all three endogenous InsP_3_R genes knocked out by CRISPR/Cas9 technology (HEK-3KO) from Kerafest (cat. # EUR030) were cultured as adherent cells in DMEM (with L-glutamine, glucose and sodium pyruvate; Corning cat. # MT10-013-CM), supplemented with 10% FBS and 1% Gibco antibiotic-antimycotic; at 37°C in 5% CO_2_. Medium was renewed every two to three days. Cells were sub-cultured following instructions provided in ATCC CRL-1573 product sheet.

HEK-3KO cells stably expressing rat InsP_3_R-3 (HEK–3KO-r–InsP_3_R–3) were generated from HEK293-3KO cells by transfecting them using Transit LT-1 (Mirus cat. # MIR 2304) following manufacturer’s protocols. Using G418 selection, stable clones of HEK293-3KO-r-InsP_3_R-3 cells expressing various level of recombinant r-InsP_3_R-3 were generated by limiting dilution.

Mouse N2a cells from ATCC (CCL-131) were cultured as adherent cells in 1:1 mixture of DMEM (high glucose with L-glutamine; Gibco cat. # 11965–092) and Opti-MEM I Reduced Serum Medium (Gibco cat. # 31985–070), supplemented with 5% FBS, 1% penicillin-streptomycin; at 37°C in 5% CO_2_. Cells were sub-cultured following instructions provided in ATCC CCL-131 product sheet.

Rat PC12 cells from ATCC (CRL-1721) were cultured as adherent cells in F-12K Kaighn’s Mod. Nutrient mixture (Corning Catalog # 10–025-CV), supplemented with 15% horse serum, 2.5% FBS, 1% Gibco antibiotic-antimycotic; at 37°C in 5% CO_2_. Cells were sub-cultured following instructions provided in ATCC CRL-1721 product sheet.

Human HEKtsA201 cells derived from HEK293 cells were obtained from Sigma-Aldrich (cat. # 96121229) and cultured as adherent cells in the same medium as for HEK293 cells, at 37°C in 5% CO_2_. 70–80% sub-confluent cells were dislodged using 0.25% trypsin solution and split 1:4 to 1:8 as described in ECACC data sheet for HEKtsA201 cells.

A549 human carcinoma cells from ATCC (CCL-185) were cultured using the same protocol as that for HEKtsA201 and HEK293 cells.

Panc-1 human pancreatic epithelioid carcinoma cells from ATCC (CRL-1469) were cultivated as adherent cells in DMEM with L-glutamine, glucose and sodium pyruvate (Corning cat. # MT10-013-CM), supplemented with 10% FBS and 1% Gibco antibiotic-antimycotic; at 37°C in 5% CO_2_. Cells were sub-cultured following instructions provided in ATCC CCL-1469 product sheet.

### Nuclear patch-clamp electrophysiology

Isolation of intact nuclei from cells, performing nuclear patch-clamp experiments in on-nucleus, cytoplasmic-side-out and luminal-side-out configurations with rapid exchange of ligand conditions are described in detail in Mak et al., 2013b; Mak et al., 2013c; Vais et al., 2010b. All solutions used for nuclear patch-clamp experiments contained 140 mM KCl, 10 mM HEPES (pH 7.3). Solutions on the cytoplasmic side of the nuclear membrane contained 2 μM free [Ca^2+^] ([Ca^2+^]_free_). [Ca^2+^]_free_ was buffered either by 0.5 mM 5,5′-dibromo 1,2-bis(o-aminophenoxy)ethane-N,N,N′,N′-tetraacetic acid (diBrBAPTA, Invitrogen D-1211 or Santa Cruz Biotechnology sc-2273516) in experiments in which [Ca^2+^]_free_ on the luminal side of the membrane ≤300 μM; or by 5 mM diBrBAPTA or 10 mM N-(2-Hydroxyethyl)ethylenediaminetriacetic acid (HEDTA, Sigma H7154) in experiments with luminal [Ca^2+^]_free_ ≥300 μM to eliminate possible permeant Ca^2+^ feed-through effects on InsP_3_R channel activities (Vais et al., 2012). Cytoplasmic solutions also contained 0.5 mM Na_2_ATP (Mak et al., 2001) and either sub-saturating 3 μM or saturating 10 μM [InsP_3_] (Vais et al., 2012) (Invitrogen I-3716 or Santa Cruz Biotechnology sc-201521) to stimulate InsP_3_R channel gating. The luminal solution with 70 nM [Ca^2+^]_free_ was buffered by 0.5 mM BAPTA; that with 2 μM [Ca^2+^]_free_ was buffered by 0.5 mM diBrBAPTA; that with 55 μM [Ca^2+^]_free_ was buffered by 0.5 mM nitrilotriacetic acid (NTA, Sigma 72559) (Dweck et al., 2005). [Ca^2+^]_free_ in these solutions were verified using [Ca^2+^]-sensitive dye fluorimetry. The luminal solution with 300 μM [Ca^2+^]_free_ was buffered by 1.5 mM Na_2_ATP according to Max Chelator freeware; and that with 600 μM [Ca^2+^]_free_ was made using the activity coefficient of CaCl_2_ as described in Vais et al., 2010a. In the experiments involving MgATP to energize the SERCA, we used a solution containing 140 mM KCl, 10 mM K-HEPES, 0.5 mM K-BAPTA, 60 μM CaCl_2_, 1 mM MgATP, pH 7.3. Using the online Webmaxc Extended software, we calculated that our solution at 25°C has 0.30 mM free Mg^2+^ (with 73 nM Ca^2+^_free_, 0.30 mM ATP^4–^, and 0.42 mM free BAPTA).

The nuclear patch-clamp experiments in various patch-clamp configurations (on-nuc, cyto-out and lum-out) using DT40-r3 cells were acquired over an extensive period. To ensure the behaviors of these cells were consistent, we regularly used cells, cultured in identical conditions and at most one day apart from data-generating cells, to perform control experiments in which nuclei were patched in on-nuc or cyto-out configurations under optimal stimulatory conditions (with 10 μM InsP_3_, 2 μM [Ca^2+^]_i_ and 0.5 mM ATP^4-^ on the cytoplasmic side of the outer nuclear membrane). Our experience suggested that most newly-thawed DT40-r3 cells were consistent after culturing for 5 days. If the mean channel *P*_o_ of a batch of control cells cultured for any time beyond day five was found to be significantly lower than that of previous control cells, that batch was terminated immediately and a new batch of cells was thawed out for new experiments.

InsP_3_R channel-current traces under constant or ramping applied potential (*V*_app_) were acquired at room temperature (RT) as described (Mak et al., 2007), digitized at 5 kHz, anti-aliasing filtered at 1 kHz. All *V*_app_ were measured relative to the reference bath electrode regardless of the patch-clamp configuration used. InsP_3_R channel gating characteristics—number of active channels observed (*N*_A_) and open probability (*P*_o_)—were derived from current traces using semi-automatic QuB (Qin et al., 2000) and fully-automatic (Bruno et al., 2013) software and manually using IGOR Pro software (Wavemetrics). Only current traces long enough for *N*_A_ to be determined with >99% confidence (Vais et al., 2010b) were used for statistical analysis.

### Ca^2+^-dependent affinity-enrichment mass spectrometry

#### Obtaining samples of enriched peripheral ER-luminal proteins from bovine hepatocytes

Bovine liver was procured from three cows within three hours of slaughter, cut into large pieces of ~200 g each, rinsed with ice-cold PBS and flash frozen in –80°C freezer within one hr of procurement to be used later. The following protocol was used for processing 200 g of bovine liver. Quantities of reagents used were scaled according to amount of extracted proteins required. Bovine liver was thawed in ice-cold water and diced into small pieces of 0.8 cm^3^ (~0.5 g). 10 g of cut liver pieces and 19 ml of ice-cold DPBS with Mg^2+^ and Ca^2+^ (Corning cat # 21–030 CM) were put into each of twenty Stomacher 80 strainer bags (Seward cat # BA6040/STR) and processed individually in a Stomacher 80 Biomaster (Seward cat # 0080/000/AJ) at highest setting for 75 s to release intact hepatocytes. Cell suspension was strained through the strainer bags and collected (~500 ml). 250 ml of ACK lysing buffer (Quality Biological cat. # 118-156-101) at RT was mixed into the cell suspension to specifically lyse red blood cells. The process was terminated after 5 min by adding 500 ml DMEM (Corning cat. # 10–013-CV) with 10% FBS followed with gentle shaking. Hepatocyte suspension was centrifuged at 9,600 *g* for 18 min and the supernatant was discarded. Hepatocytes were rinsed twice by resuspending cells in 500 ml HBSS, centrifuging at 9,600 *g* for 18 min, and discarding the supernatant.

Hepatocytes were resuspended in 150 ml of homogenizing solution containing 150 mM KCl, 1 mM MgCl_2_, 1 mM CaCl_2_, 20 mM Tris HCl (pH 7.5), 400 μM phenylmethane sulfonyl fluoride (PMSF), and seven cOmplete protease inhibitor cocktail tablets (Roche cat. # 11697498001). Debris in cell suspension was removed with cell strainer. The hepatocytes were homogenized with nitrogen cavitation in an ice-cold large cell disruption vessel (Parr Instrument, Moline Il, model # 4635) using pressure of 1000 p.s.i. with constant stirring for 20 min. Cell homogenate was centrifuged at 3,300 *g* for 15 min to pellet unbroken cells and nuclei. The supernatant was centrifuged at 9,600 *g* for 15 min to pellet cell debris and mitochondria, and at 105,000 *g* for 15 min to pellet a crude microsomal fraction. The pellet was resuspended into 40 ml of 1.38 M sucrose solution, placed under a step gradient of 1.0 and 0.86 M sucrose and centrifuged at 300,000 *g* for 75 min. The smooth and rough ER fractions below the interface between 1.38 M and 1.0 M sucrose solution layers were collected and placed on ice.

To remove ribosomes from the rough ER, ice-cold sodium pyrophosphate (NaPP_i_)-imidazole HCl (NaPP_i_-I) solution containing 5 mM NaPP_i_ and 3 mM imidazole-HCl, pH 7.4 was added dropwise to the collected ER fractions with continuous stirring on ice to the final volume of 100 ml. After a further 15 min of stirring, the mixture was centrifuged at 105,000 g for 37 min to re-pellet the microsomes. After discarding the supernatant, the ER microsome pellet was resuspended in 45 ml of sucrose-NaPP_i_-I solution containing 0.25 M sucrose, 5 mM NaPP_i_, and 3 mM imidazole HCl, pH 7.4; centrifuged again at 105,000 *g* for 37 min; and the supernatant was discarded.

The smooth ER microsome pellet was resuspended with vigorous vortexing in 8 ml of low-Ca^2+^ K^+^ with protease inhibitor (LCaK-PI) solution containing 150 mM KCl, 0.5 mM EGTA, 0.05% Tween-20, 25 mM Tris HCl (pH 7.5), 400 μM PMSF, one cOmplete protease inhibitor cocktail mini-tablet (Roche cat. #11836170001). The microsome suspension was processed with nitrogen cavitation twice using an ice-cold small cell disruption vessel (Parr Instrument, Moline Il, model # 4639) under 2200 p.s.i. with constant stirring for 20 min each cavitation. The homogenate was centrifuged at 105,000 *g* for 60 min to pellet the broken ER membrane. The supernatant was collected carefully without disturbing the ER membrane pellet so it contained only peripheral proteins released from the ER lumen in the presence of low [Ca^2+^]_free_ (buffered by 0.5 mM EGTA in the LCaK-PI solution). Total protein concentration in the hepatocyte peripheral ER luminal protein (HPERLP) sample was determined using Bradford protein assay (~1.4–3.0 mg/ml). The HPERLP sample was aliquoted and stored in –80°C for future use.

#### Magnetic-bead pull down of peripheral proteins in the ER lumen that specifically interact with the luminal region of the InsP_3_R

Based on the pL2 sequence in the L2 region of the InsP_3_R that is highly conserved among the three InsP_3_R isoforms (Figure 5B), peptides with a modified sequence (mpL2: *K*-Ahx-GNRGTFIRGYRAMVMDME) were synthesized for pull-down experiments. The Lys residue in pL2 was replaced with Arg (underlined) so that the mpL2 peptides could be coupled to magnetic beads through the only Lys (in italics) at the N-terminus. The unnatural 6-aminohexanoate residue (Ahx) was added to reduce possible steric hindrance that may interfere with the interaction between the peptide and ER luminal proteins. For control, peptides with a scrambled modified sequence (smpL2: *K*-Ahx-RDYRGMRMIMGETFNVGA) were also synthesized. Instructions of the manufacturer were followed to covalently couple the mpL2 and smpL2 peptides to NHS-activated magnetic beads (Pierce cat. # 88826) through the stable covalent link formed by reaction between the primary amine group of the N-terminal Lys of the peptides and the *N-*hydroxysuccinimide ester group on the magnetic beads. Peptide-coupled beads were stored (10 mg/ml) in storage buffer (150 mM NaCl, 25 mM Tris, pH 7.4 with 0.05% Tween-20% and 0.05% sodium azide) at 4°C.

A total of six pull-down procedures were performed in the cold with all apparatus and reagents at 4°C: one procedure using mpL2 peptide-coupled magnetic beads and one control procedure using smpL2 peptide-coupled magnetic beads for each of the three HPERLP samples from individual cows. The same protocol described below was used for each of the six procedures. To obtain sufficient protein for mass spectrometry, eight MACS separation columns (MS columns, Miltenyi Biotec cat. # 130-042-201) were used in each procedure. The MS columns were placed on the OctoMACS separator (Miltenyi Biotec cat. # 130-042-109) mounted on the MACS Multistand (Miltenyi Biotec cat. #130-042-303). To prime the columns, 250 μl of high-Ca^2+^ K^+^ (HCaK) solution containing 150 mM KCl, 1% NP–40 detergent (Thermo scientific cat. # 85124), 0.8 mM CaCl_2_, 25 mM Tris HCl (pH 7.5) was added to each. Effluent from the columns was discarded. 320 μl of peptide-coupled magnetic beads were resuspended in storage buffer, rinsed twice with HCaK solution, and resuspended with 290 μl of HCaK solution. 35 μl of the peptide-coupled magnetic bead suspension was added to each of the MS columns. Effluent was examined for presence of magnetic beads. If beads were detected, effluent was returned to the column till no beads were detected. Each column with magnetic beads were washed with 80 μl of HCaK solution and effluent was discarded. 360 μl of the HPERLP sample was thawed and HCaK solution and 1 M CaCl_2_ standard solution were added to adjust the total protein concentration in the final HCaK-HPERLP mixture to 1.25 mg/ml, and [CaCl_2_] to 800 μM ([Ca^2+^]_free_ = 432 μM). 40 μl of the HCaK-HPERLP mixture was added to each column, followed by 80 μl of HCaK-PI solution—HCaK solution with additional 400 μM PMSF and 1% Halt protease inhibitor cocktail (Thermo Fisher Cat. # 78430). The magnetic beads in each column were rinsed once with 2 ml of HCaK-PI solution, and thrice with 2 ml of HCaK solution. Effluent was discarded. 200 μl of low-Ca^2+^ eluding (LCaE) solution with 150 mM KCl, 1% NP–40 detergent, 0.5 mM EGTA, 25 mM Tris HCl (pH 7.5) was added to each column. The eluate from all the columns was collected and frozen at –80°C.

#### Loading samples into mass spectrometer

Low-Ca^2+^ eluate proteins from above were precipitated with 5 volumes of acetone held overnight at –20°C, decanted then solubilized in 20 µL of 1 × reducing NuPAGE LDS (Thermo Fisher Scientific) and run into a precast 10% Bis-Tris SDS-PAGE (Thermo Fisher Scientific) for 1.6 cm. After fixing overnight (25% ethanol, 7% acetic acid) the gel was stained with colloidal Coomassie blue dye. Each sample was excised from the gel in 4 (2 × 9 mm) slices and each cut into (1 mm)^3^ cubes. The gel pieces were destained in a solution containing 50% methanol and 1.25% acetic acid, reduced with 5 mM DTT (Thermo Fisher Scientific), and alkylated with 40 mM IAA (Sigma-Aldrich). The gel pieces were washed with 20 mM ammonium bicarbonate (Sigma-Aldrich) and dehydrated with acetonitrile (Fisher Scientific) twice. Sequencing-grade trypsin (Promega) (5 ng/µL in 20 mM ammonium bicarbonate) was added to the dehydrated-gel pieces and proteolysis was allowed to proceed overnight at 37°C. Peptides were extracted with 0.3% TFA (Fisher Scientific), followed by 50% acetonitrile. The volume of the combined extracts was reduced by vacuum centrifugation and 25% of the extracted peptides were analyzed per LC-MS/MS run (Glisovic-Aplenc et al., 2017).

#### LC-MS/MS mass spectrometry

Tryptic digests were analyzed by LC-MS/MS on a hybrid LTQ Orbitrap Elite mass spectrometer (Thermofisher Scientific San Jose, CA) coupled with a nanoLC Ultra (Eksigent). Peptides were separated by reverse phase (RP)-HPLC on a nanocapillary column, 75 μm id ×15 cm Reprosil-pur 3 μm, 120A (Dr. Maisch, Germany) in a Nanoflex chip system (Eksigent). Mobile phase A consisted of 1% methanol (Fisher)/0.1% formic acid (Thermo) and mobile phase B of 1% methanol/0.1% formic acid/80% acetonitrile. Peptides were eluted into the mass spectrometer at 300 nL/min with each RP-LC run comprising a 90 min gradient from 10% to 25% B in 65 min, 25–40% B in 25 min. The mass spectrometer was set to repetitively scan m/z from 300 to 1800 (R = 240,000 for LTQ-Orbitrap Elite) followed by data-dependent MS/MS scans on the twenty most abundant ions, with a minimum signal of 1500, dynamic exclusion with a repeat count of 1, repeat duration of 30 s, exclusion size of 500 and duration of 60 s, isolation width of 2.0, normalized collision energy of 33, and waveform injection and dynamic exclusion enabled. FTMS full scan AGC target value was 1e6, while MSn AGC was 1e4, respectively. FTMS full scan maximum fill time was 500 ms, while ion trap MSn fill time was 50 ms; microscans were set at one. FT preview mode; charge state screening, and monoisotopic precursor selection were all enabled with rejection of unassigned and 1+ charge states.

#### Sequence database search

Raw MS files were processed using MaxQuant, version 1.6.1.0 for identification of proteins. The peptide MS/MS spectra were searched against the UniProtKB/Swiss-Prot Bovine Reference Proteome database, UP000009136 (retrieved on 12 July, 2018), comprising 24,333 entries including isoforms. Fragment ion tolerance was set to 0.5 Da, with full tryptic specificity required and a maximum of two missed tryptic cleavage sites. Precursor ion tolerance was seven ppm. Oxidation of methionine, acetylation of the protein N-terminus and conversion of glutamine to pyroglutamic acid were used as variable modifications. Carbamidomethylation of cysteine was set as a fixed modification. The minimal length required for a peptide was seven amino acids. Target-decoy approach was used to control false discovery rate (FDR). A maximum FDR of 1% at both the peptide and the protein level was allowed. If no unique peptide sequence to a single database entry was identified, the resulting protein identification was reported as an indistinguishable ‘protein group’. Protein groups containing matches to decoy database or contaminant proteins were discarded. The MaxQuant match-between-runs feature was enabled.

#### Statistical analysis of mass spectrometry output

We modeled the MS/MS spectral counts for each protein, sample pair using a hierarchical Poisson log-linear model. Our Bayesian method helped circumvent some of the problems that arise with small sample sizes by borrowing strength across proteins to better estimate the parameters in the model. We performed inference to determine the proteins that preferentially bound to the bait peptide found in InsP_3_R compared to the scrambled, control peptide using the false sign rate paradigm (Stephens, 2017).

Let $y_{gct}y_{gct}$ be the number of observed MS/MS spectra for protein g=1,...p from cow c=1,...,Cc=1,...,C in sample t∈{0,1}t∈{0,1}, where *t*t is 0 if the bait molecule was the scrambled peptide and one if the bait molecule was the modified InsP_3_R L2, that is a treated sample. In these data, C=3C=3 and *p*=486p=486. For $\delta_{0}$ a point mass at 0, we model the data as follows:

$$\begin{array}{l} y_{gct}(\lambda_{gct})+1∼Poi(\lambda_{gct})\;\;(g=1,...,p;c=1,...C;t=0,1)\\ log(\lambda_{gct})=log(\mu_{c})+\alpha_{gc}+t\beta_{g}\;\;(g=1,...,p;c=1,...C;t=0,1)\\ \alpha_{gc}∼N(0.\sigma_{c}^{2})\;\;(g=1,...,p;c=1,...,C)\\ \beta_{g}∼\pi_{0}\delta_{0}+(1-\pi_{0})N(0,\tau^{2})\\ \pi_{0}∼Beta(0.5,0.5)\\ \sigma_{c}^{-2},.\tau^{-2}∼Gamma(1,1)\;\;\;(c=1,...,C) \end{array}$$

and set $\mu_{c}=p^{-1}\Sigma_{g=1}^{p}\frac{y_{gc0}+y_{gc1}+2}{2}\mu_{c}=p^{-1}\Sigma_{g=1}^{p}\frac{y_{gc0}+y_{gc1}+2}{2}$. We added a pseudo count of 1 spectral count to each data point to avoid taking the log of 0. Note that adding a pseudo count only makes it more challenging to identify differences between the treatment and control conditions, and therefore leads to conservative inference. The above model also helped mitigate latent confounding variables and sources of over-dispersion by explicitly accounting for between-cow variation.

The goal was to estimate and perform inference on the treatment effect $\beta_{g}$ for each protein g=1,...,p,, where we were interested in identifying proteins with $\beta_{g}>0$ because these were indicative of proteins that were comparatively enriched in the treated, experimental samples. The local false sign rate for each protein is defined as the posterior probability that protein *g* did not preferentially bind to the InsP_3_R peptide:

$$lfsr_{g}=P(\beta g\leq 0|Y)\;\;(g=1,...,p).$$

The local false sign rates were computed by sampling from the posterior using Markov Chain Monte Carlo. A low LFSR_G_ value indicates that the posterior probability of incorrectly identifying a protein as being enriched in the experimental samples is small. A threshold of LFSR_G_ ≤ 0.2 (corresponding to a global false discovery rate of 5%) was applied to identify proteins with strong preference to bind to the L2 region of the InsP_3_R.

### Confocal microscopy

Images were collected on a Leica TCS SP8 X system using a 100x/1.4 NA PL APO CS2 objective. Annexin A1 was labeled with rabbit anti-Annexin A1 pAb (Proteintech, 21990–1-AP) and anti-rabbit AlexaFluor568 conjugated pAb (Invitrogen, A11011) was excited using a continuously tunable white light laser set to 568 nm to detect fluorescence emission between 600–700 nm. Calnexin was labeled with mouse anti-Calnexin mAb (Chemicon, MAB3126) and anti-mouse AlexaFluor488 conjugated pAb (Invitrogen, A11001) excited at 488 nm to detect fluorescence emission between 510–550 nm. DAPI was present in the mounting medium used to prepare the slides, excited at 408 nm to detect fluorescence emission between 430–470 nm.

### Capture ELISA to monitor secretion of ANXA1 from HEKtsA201 cells

#### Sample preparation

HEKtsA201 cells were seeded at 2 × 10^6^ cells/10 cm tissue culture dish in 10 ml of 5% FBS/DMEM with 1 × Anti Anti, and grown at 37°C in 5% CO_2_ for 72 hr. Media in the dishes was collected, filtered through a 0.22 µm pore syringe filter, and concentrated 8-fold using Amicon Ultra-4 centrifugal filter devices (3 kDa MW-cutoff) as described in the manufacturer’s protocol. The cells in each dish were detached with Versene (5.4 mM EDTA) solution, pelleted by centrifugation at 1000 RPM for 5 min at 4°C, rinsed in 10 ml of 1 × DPBS, pelleted, and re-suspended in 1.5 ml of cell lysis buffer (50 mM Tris-HCL, 150 mM NaCl, 0.5 mM EGTA-NaOH, 0.3 mM CaCl_2_, and 0.2% w/v n-dodecyl β-d-maltoside) supplemented with 1 mM PMSF and Protease Inhibitor Cocktail (Roche). Lysates were incubated for 15 min on ice, centrifuged at 14,000 RPM for 10 min at 4°C to pellet genetic material released from nuclei. Supernatants were collected. Total [protein] was determined using the Pierce BCA Protein Assay kit (Thermo Fisher cat. # 23227).

#### Capture ELISA

A high-binding, white, 96-well microtiter plate (Thermo, 7572) was coated with 100 µl/well of rabbit α-ANXA1 antibody (Proteintech, 21990–1-AP) diluted 1:500; or rabbit α-β actin antibody (Cell Signaling Technologies, 7881S) diluted 1:100 in carbonate capture buffer (6 mM Na_2_CO_3_; 44 mM NaHCO_3_; pH 9.6). Plates were incubated overnight at 4°C. The next day, the capture antibody solution was discarded, wells were rinsed 3 × with 200 µl of wash solution (50 mM Tris, 0.14 M NaCl, 0.05% Tween 20, pH 8.0), and 200 µl of blocking solution (50 mM Tris, 0.14 M NaCl, 1% BSA, pH 8.0) was added to each well and incubated for 30 min at RT. The wells were rinsed 3 × again as described before. 100 µl of each concentrated cell-culture media (supernatant) or cell lysate sample were added. Five-fold serial dilutions were made of concentrated supernatants (neat, 1:5, 1:25, 1:125 in cell culture media) and cell lysates (0.5, 0.1, 0.02, 0.004, 0.0008 mg/mL in cell lysis buffer) in six replicates. Wells containing cell-culture media or cell lysis buffer only were made in triplicate as negative controls. The plate was incubated overnight at 4°C. The next day, samples in the wells were discarded, and the wells rinsed 3×. 100 µl of primary mouse α-ANXA1 antibody (ECM Biosciences, AM0211) diluted 1:1000; or mouse α-pan actin antibody (Cell Signaling Technologies, 7881S) diluted 1:100 in antibody diluent (50 mM Tris, 0.14 M NaCl, 1% BSA, 0.05% Tween 20, pH 8.0) was added to the appropriate wells and incubated for 1 hr at RT. Contents in the wells were again discarded and the wells rinsed 3×. 100 µL of secondary goat α-mouse IgG-HRP antibody (Cell Signaling Technologies, 7074S) diluted 1:2000 in antibody diluent was added to each well, and incubated for 1 hr at RT. Again, contents in the wells were discarded and the wells rinsed 3×. 100 µL of PICO chemiluminescent HRP substrate (Pierce) was added to each well and incubated for 5 min at RT. Chemiluminescence was measured in a Cytation five plate reader (BioTek).

### InsP_3_R-mediated intracellular Ca^2+^ release in HEKtsA201 cells expressing different levels of ANXA1

#### Acute knock-down of ANXA1 expression in HEKtsA201 cells by siRNA transfection

We performed knock-down experiments by transfecting human HEKtsA201 cells with human ANXA1 siRNA. For comparison, two kinds of controls were performed: HEKtsA201 cells were either transfected with non-targeting (N-T) siRNA or not transfected at all. Four pairs of controls and experiments were done: two pairs were experiments using ANXA1 siRNA-transfected cells versus controls using N-T siRNA-transfected cells, the other two were experiments using ANXA1 siRNA-transfected cells versus controls using non-transfected cells.

Following the siRNA transfection protocol from Dharmacon (Lafayette, CO), human ANXA1 siRNA (Dharmacon cat. # M-011161-01-0005) and N-T siRNA (Dharmacon cat. # D-001206-13-05) were suspended in 250 μl of 1 × siRNA buffer (50 μl of 5 × siRNA buffer (Dharmacon cat. # B-002000-UB-100) in 200 μl RNase-free water) to a final siRNA concentration of 20 μM, aliquoted and stored at –20°C. For a pair of control and experiment, 10 ml of HEKtsA201 cell suspension with density of ~0.26 × 10^6^ cells/ml were plated into each of four 10 cm culture dishes (two for the experiment and two for the control) and incubated overnight (~18 hr) at 37°C. To transfect two 10 cm dishes of HEKtsA201 cells, 30 μl of 20 μM siRNA (ANXA1 or N-T) was thawed and diluted with 90 μl of RNase-free water. 3.9 ml of Opti-MEM (Gibco cat. # 31985–070) was added to the siRNA, mixed by gentle pipetting. The siRNA mixture was incubated for 7 min. For a non-transfected control, 4 ml of Opti-MEM was used. 160 μl of transfection reagent (Dharmacon cat. # T-2001–02) was diluted with Opti-MEM to a final volume of 8 ml, mixed by gentle pipetting. The resulting transfection solution was incubated for 7 min. 4 ml of transfection solution was added to the 4 ml of siRNA mixture (for experiment ANXA1 or control N-T siRNA transfection) or 4 ml of Opti-MEM solution (for non-transfection control). The resulting mixtures were incubated for another 25 min. Each of the mixtures was then diluted with 32 ml of antibiotic-free complete medium (DMEM (Corning cat. # 10–013-CV) with 10% FBS (HyClone cat. # SH30071.03) to give 40 ml of transfection medium.

Culture medium was removed from each of the four dishes of HEKtsA201 cells that had been incubated overnight. 20 ml of ANXA1 siRNA transfection medium was added to each of two dishes for the experiment; and 20 ml of N-T siRNA transfection medium or non-transfection medium was added to each of two dishes for the control. The cells in transfection medium were incubated for 6 hr at 37°C. After the incubation, the transfection medium was replaced with 20 ml of regular HEKtsA201 cell medium for each of the four dishes of cells. Cells were incubated at 37°C for an additional 42 hr before being used for measurements of intracellular Ca^2+^ release.

Expression levels of ANXA1 in HEKtsA201 cells were probed in Western blots using anti-ANXA1 antibody (Proteintech cat. # 21990–1-AP, 1:1000 dilution), stripped and re-probed with anti-β-actin antibody (Cell Signalling cat. # 8H10D10, 1:1000 dilution).

#### Spectrofluorimetry of intracellular Ca^2+^ release stimulated by extracellular agonist

The same protocol was used to perform fluorimetry for experiment and control HEKtsA201 cells. After incubation,~10–20 × 10^6^ cells (ANXA1 siRNA transfected, N-T siRNA transfected or non-transfected) were in each 10 cm culture dish. To collect the attached cells, the HEKtsA201 medium was removed, the cells were rinsed once with 10 ml of Ca^2+^-free DPBS (Corning cat. # 21–031 CM) at RT, incubated in 5 ml of Versene solution (Gibco cat. # 15040–066) for 20 min to detach, dispersed by gently pipetting the cell suspension, and the cell density was determined using a hemocytometer. Four million cells were used to quantify the expression level of ANXA1 in the HEKtsA201 cells by Western blotting. The remaining cells were centrifuged at 350 *g* for 3 min. The supernatant was discarded, and the cell pellet was resuspended in 10 ml of Fura-2 AM dye-loading solution (1 μM Fura-2 AM dye (Molecular Probes cat. # I-1225) and 0.01% pluronic F127 (Thermo Fisher cat. # P3000MP) in HEPES-buffered saline (HBS) containing 135 mM NaCl, 5.9 mM KCl, 1.2 mM MgCl_2_, 1.5 mM CaCl_2_, 11 mM glucose, and 11.6 mM Na-HEPES (pH 7.4). The cell suspension was kept in darkness with gentle shaking for 20 min at RT. The dye-loaded cells were then centrifuged at 350 *g* for 3 min. The supernatant was discarded, and the cell pellet was resuspended in 10 ml HBS. The cell suspension was again centrifuged at 350 *g* for 3 min. The supernatant was discarded, and the cell pellet was resuspended in HBS to a cell density of 3–4 × 10^6^ cells/ml. These cells were kept in darkness. Each 10 cm dish of cells was sufficient for 4 to 7 assays. For proper control, dishes of ANXA1 siRNA-transfected cells and control cells were used alternately. Just before each assay, 0.75 ml of cell suspension was centrifuged in a 1.5 ml eppendorf tube at 400 *g* for 3 min. The supernatant was discarded and the cells were resuspended in 0.75 ml of HBS and transferred to a cuvette containing 0.75 ml of HBS and a 3–mm magnetic stirrer. The cuvette was transferred to the temperature-controlled (37°C) experimental compartment of a multi-wavelength-excitation dual-wavelength-emission high-speed spectrofluorometer (Delta RAM, Photon Technology International). Fluorescence at 340 nm excitation/515 nm emission and 380 nm excitation/515 nm emission for the Ca^2+^-bound and Ca^2+^-free Fura-2 species, respectively, were measured at 5 Hz. The ratio (*R*) of the fluorescence intensity excited by 340 nm versus 380 nm light was used as relative measurement of intracellular [Ca^2+^]_free_. After a stable baseline of *R* had been observed for 90 s, 15 μl of 10 mM carbachol solution was added to the cell suspension (final 10 μM carbachol) to stimulate sub-maximal InsP_3_R-mediated release of Ca^2+^ from the ER. *R* was further observed for another 120 s.

### Imaging histamine-induced Ca^2+^ signals in HeLa cells expressing different levels of ANXA1

HeLa cells were cultured on 15 cm tissue culture dishes in Dulbecco’s Modification of Eagle’s (DMEM Corning) supplemented with 10% fetal bovine serum (Hyclone) and maintained at 37°C in a humidified incubator gassed with 95% air and 5% CO_2_. Transfection with siRNA for either human *ANXA1* siRNA or non-targeting siRNA control was performed as described for HEKtsA201 cells. ~ 3 × 10^5^ cells were seeded on each 12 mm coverslip. At 48 hr post-transfection, cells were loaded with 2 µM Fura-2 AM for 15–20 min then incubated in 2 Ca Tyrode’s solution for 5–10 min at room temperature prior to imaging. Cells were imaged on a Nikon Ti system using a 20x/0.75 NA objective for fluorescence at 340 nm excitation/515 nm emission (Ca^2+^-bound Fura2), and 380 nm excitation/515 nm emission (Ca^2+^-free Fura2). Coverslips were perfused with 2 Ca Tyrode’s solution for 30 s, 10 or 100 µM histamine in 2 Ca Tyrode’s solution for 360 s or 120 s respectively, followed by a washout with 2 Ca Tyrode’s solution for 90 s. 100 ms exposure images for each wavelength were collected every 2 s. For analysis of intracellular Ca^2+^-signals, cells were mass-selected using a binary mask and the Time Series Analyzer v3 plugin in ImageJ (NIH). The 340 nm/380 nm (R_fura2_) fluorescence ratio for each time point was measured for every cell in the field of view and the normalized signal (R/R_0_) was calculated by dividing R_fura2_ for each time point by the mean R_fura2_ during the first 30 s of each experiment. A series of logic tests were applied to data to identify signaling cells and separate those cells into oscillating and non-oscillating groups. To exclude cells that did not begin and end at baseline Ca^2+^ levels, only cells with R/R_0_ values between 1.25–0.75 during the first 30 s and R/R_0_ values less than 1.5 during the last 30 s of the final washout were selected. To exclude non-responding cells, only cells with maximal R/R_0_values ≥ 2.0 following histamine stimulation were selected. Cells with a coefficient of variation (%CV) value greater than 15% measured from the end of the initial Ca^2+^ signal to the end of the histamine stimulation were considered oscillating cells. Oscillating cells were qualitatively characterized as exhibiting either ‘periodic oscillation’ or ‘random spiking’ pattern.

### Monitoring intracellular elementary Ca^2+^ release events (Ca^2+^ puffs) in HEK293 cells by total internal reflection microscopy

#### Cell culture and siRNA transfection

WT HEK-293 cells were cultured on plastic 75 cm tissue culture flasks in Eagle’s Minimum Essential Medium (EMEM; ATCC #30–2003) supplemented with 10% fetal bovine serum (Omega Scientific #FB-11), and maintained at 37°C in a humidified incubator gassed with 95% air and 5% CO_2_. For transfection with siRNA, cells were collected using 0.25% Trypsin-EDTA (Gibco #25200–056) and grown on 60 mm culture dishes. When cells reached 80% confluence they were incubated with 15 nM *ANXA1* siRNA or with 15 nM non-targeting CTL siRNA together with 15 µl of DharmaFECT transfection reagent (GE Healthcare) for 6 hr. In some experiments, cells were additionally incubated with 5 nM siGLO Red Transfection Indicator (Dharmacon #D-001630-02-05) to serve as a fluorescent tracer to visualize transfection efficiency. Following incubation, cells were harvested using 0.25% Trypsin-EDTA, pelleted by low speed centrifugation (~800 x g), resuspended in EMEM and distributed among poly-D-lysine coated (1 mg/ml; Sigma #P0899) 35 mm glass-bottom imaging dishes (MatTek #P35-1.5–14 C) where they were grown for 48 hr prior to imaging, at which time cells were ~50% confluent.

Immediately before imaging, cells were incubated with membrane-permeable esters of the fluorescent Ca^2+^ dye Cal-520/AM (5 µM; AAT Bioquest #21130) and the caged IP_3_ analogue ci-IP_3_/PM [D-2,3,-O-Isopropylidene-6-O-(2-nitro-4,5 dimethoxy) benzyl-myo-Inositol 1,4,5,-trisphosphate Hexakis (propionoxymethyl) ester] (1 µM; SiChem #cag-iso-2-145-10) for 1 hr at RT in a Ca^2+^-containing HEPES buffered salt solution (Ca^2+^-HBSS). Cells were then washed with Ca^2+^-HBSS and loaded for an additional hour with EGTA/AM (5 µM; ThermoFisher #E1219). Cal-520/AM, caged i-IP_3_/PM, and EGTA/AM were all solubilized with DMSO/20% pluronic F127 (ThermoFisher #P3000MP). Ca^2+^-HBSS contained (in mM) 135 NaCl, 5.4 KCl, 2 CaCl_2_, 1 MgCl_2_, 10 HEPES, and 10 glucose (pH = 7.4 at RT).

#### Ca^2+^ imaging

Total internal reflection fluorescence (TIRF) imaging of Ca^2+^ signals was accomplished using a home-built system, based around an Olympus IX50 microscope equipped with an Olympus 60X objective (NA 1.45). Using 488 nm laser fluorescence excitation and a 510 nm long pass emission filter, fluorescence images were captured with an Evolve EMCCD camera (Photometrics), utilizing 2 × 2 pixel binning for a final field of 128 × 128 pixels (one pixel = 0.53 µm) at a rate of ~125 frames s^−1^. To photo-release i-IP_3_, UV light from a xenon arc lamp was filtered through a 350–400 nm bandpass filter and introduced by a UV-reflecting dichroic in the light path to uniformly illuminate the field of view. The amount of i-IP_3_ released was controlled by varying the flash duration, set by an electronically controlled shutter (UniBlitz). All image data were streamed to computer memory using Metamorph v7.7 (Universal Imaging/Molecular Devices) and stored on hard disc for offline analysis.

#### Image analysis

Image data in MetaMorph stk format were processed using Flika (Ellefsen et al., 2014), a freely available open-source image processing and analysis software written in the Python programming language (Ellefsen et al., 2019). Fluorescence records of 30 s and 10 s immediately following the UV flash were analyzed for the 50 ms and 150 ms flash durations, respectively; shorter records were analyzed for the 150 ms flash duration because of rapidly rising global Ca^2+^ levels which hamper measurement of local Ca^2+^ signals. Raw fluorescence records were first black-level subtracted and then processed to create a ratio image stack (F/F_0_), where the fluorescence intensity of each pixel in a given frame was replaced by that intensity (F) divided by the mean resting fluorescence intensity at that pixel averaged over 500 frames prior to photo-release of i-IP_3_ (F_0_). Next, a custom plug-in (‘detect puffs’) was applied for automated detection and analysis of local Ca^2+^ signals. All Ca^2+^ puffs identified by the algorithm were verified by visual inspection prior to further analysis. Measurement of peak puff amplitudes (ΔF/F_0_) and kinetics were performed by the algorithm on a 5 × 5 pixel region of interest centered over the centroid of each event, and were exported to EXCEL spreadsheets for further analysis. Additional analysis and graphing was performed in Microcal Origin v6.0 (OriginLab) and Igor 6 (WaveMetrics).

### Quantification and statistical analyses

All measurements in this study: InsP_3_R channel open probabilities (*P*_o_), normalized probabilities of detection of InsP_3_R channel activity (*P*_d_), normalized maximal change of fluorescence intensity ratio (Δ*R*_max_) and normalized rate of change of fluorescence intensity (1/*τ*), number of Ca^2+^ puffs per second per imaging field and amplitudes of the Ca^2+^ puffs (ΔF/F_0_), performed under various experimental conditions were compared using unpaired *t*-test. For data sets involved in multiple comparisons, *p* values were determined using the Bonferroni correction for multiple comparisons. Quantified western blot intensities from co-immunoprecipitation experiments were compared by paired *t*-test.

## Data Availability

All data generated and analyzed are included in the manuscript and supporting files.

## Additional files

Transparent reporting form

## References

[bib1] Arur S, Uche UE, Rezaul K, Fong M, Scranton V, Cowan AE, Mohler W, Han DK (2003). Annexin I is an endogenous ligand that mediates apoptotic cell engulfment. Developmental Cell.

[bib2] Belvedere R, Bizzarro V, Popolo A, Dal Piaz F, Vasaturo M, Picardi P, Parente L, Petrella A (2014). Role of intracellular and extracellular annexin A1 in migration and invasion of human pancreatic carcinoma cells. BMC Cancer.

[bib3] Berridge MJ (2016). The inositol trisphosphate/Calcium signaling pathway in health and disease. Physiological Reviews.

[bib4] Boudhraa Z, Bouchon B, Viallard C, D'Incan M, Degoul F (2016). Annexin A1 localization and its relevance to Cancer. Clinical Science.

[bib5] Brownstein C, Falcone DJ, Jacovina A, Hajjar KA (2001). A mediator of cell surface-specific plasmin generation. Annals of the New York Academy of Sciences.

[bib6] Bruno WJ, Ullah G, Mak DO, Pearson JE (2013). Automated maximum likelihood separation of signal from baseline in noisy quantal data. Biophysical Journal.

[bib7] Caroppo R, Colella M, Colasuonno A, DeLuisi A, Debellis L, Curci S, Hofer AM (2003). A reassessment of the effects of luminal [Ca^2+^] on inositol 1,4,5-trisphosphate-induced Ca^2+^ release from internal stores. The Journal of Biological Chemistry.

[bib8] Chi A, Valencia JC, Hu ZZ, Watabe H, Yamaguchi H, Mangini NJ, Huang H, Canfield VA, Cheng KC, Yang F, Abe R, Yamagishi S, Shabanowitz J, Hearing VJ, Wu C, Appella E, Hunt DF (2006). Proteomic and bioinformatic characterization of the biogenesis and function of melanosomes. Journal of Proteome Research.

[bib9] Corbett EF, Michalak M (2000). Calcium, a signaling molecule in the endoplasmic reticulum?. Trends in Biochemical Sciences.

[bib10] D'Acquisto F, Merghani A, Lecona E, Rosignoli G, Raza K, Buckley CD, Flower RJ, Perretti M (2007). Annexin-1 modulates T-cell activation and differentiation. Blood.

[bib11] Dingwall C, Laskey R (1992). The nuclear membrane. Science.

[bib12] Dweck D, Reyes-Alfonso A, Potter JD (2005). Expanding the range of free calcium regulation in biological solutions. Analytical Biochemistry.

[bib13] Ellefsen KL, Settle B, Parker I, Smith IF (2014). An algorithm for automated detection, localization and measurement of local calcium signals from camera-based imaging. Cell Calcium.

[bib14] Ellefsen KL, Lock JT, Settle B, Karsten CA, Parker I (2019). Applications of FLIKA, a Python-based image processing and analysis platform, for studying local events of cellular calcium signaling. Biochimica Et Biophysica Acta (BBA) - Molecular Cell Research.

[bib15] Fan X, Krahling S, Smith D, Williamson P, Schlegel RA (2004). Macrophage surface expression of annexins I and II in the phagocytosis of apoptotic lymphocytes. Molecular Biology of the Cell.

[bib16] Fan G, Baker ML, Wang Z, Baker MR, Sinyagovskiy PA, Chiu W, Ludtke SJ, Serysheva II (2015). Gating machinery of InsP_3_R channels revealed by electron cryomicroscopy. Nature.

[bib17] Foskett JK, White C, Cheung KH, Mak DO (2007). Inositol trisphosphate receptor Ca^2+^ release channels. Physiological Reviews.

[bib18] Gerke V, Moss SE (2002). Annexins: from structure to function. Physiological Reviews.

[bib19] Glisovic-Aplenc T, Gill S, Spruce LA, Smith IR, Fazelinia H, Shestova O, Ding H, Tasian SK, Aplenc R, Seeholzer SH (2017). Improved surfaceome coverage with a label-free nonaffinity-purified workflow. Proteomics.

[bib20] Higo T, Hattori M, Nakamura T, Natsume T, Michikawa T, Mikoshiba K (2005). Subtype-specific and ER lumenal environment-dependent regulation of inositol 1,4,5-trisphosphate receptor type 1 by ERp44. Cell.

[bib21] Higo T, Hamada K, Hisatsune C, Nukina N, Hashikawa T, Hattori M, Nakamura T, Mikoshiba K (2010). Mechanism of ER stress-induced brain damage by IP_(3)_ receptor. Neuron.

[bib22] Iwasa T, Takahashi R, Nagata K, Kobayashi Y (2012). Suppression of MIP-2 or IL-8 production by annexins A1 and A4 during coculturing of macrophages with late apoptotic human peripheral blood neutrophils. Biochimica Et Biophysica Acta (BBA) - Molecular Basis of Disease.

[bib23] Keebler MV, Taylor CW (2017). Endogenous signalling pathways and caged IP_3_ evoke Ca^2+^ puffs at the same abundant immobile intracellular sites. Journal of Cell Science.

[bib24] Lanini L, Bachs O, Carafoli E (1992). The calcium pump of the liver nuclear membrane is identical to that of endoplasmic reticulum. The Journal of Biological Chemistry.

[bib25] Leoni G, Neumann PA, Kamaly N, Quiros M, Nishio H, Jones HR, Sumagin R, Hilgarth RS, Alam A, Fredman G, Argyris I, Rijcken E, Kusters D, Reutelingsperger C, Perretti M, Parkos CA, Farokhzad OC, Neish AS, Nusrat A (2015). Annexin A1-containing extracellular vesicles and polymeric nanoparticles promote epithelial wound repair. Journal of Clinical Investigation.

[bib26] Lock JT, Alzayady KJ, Yule DI, Parker I (2018). All three IP_3_ receptor isoforms generate Ca^2+^ puffs that display similar characteristics. Science Signaling.

[bib27] Lock JT, Smith IF, Parker I (2019). Spatial-temporal patterning of Ca^2+^ signals by the subcellular distribution of IP_3_ and IP_3_ receptors. Seminars in Cell & Developmental Biology.

[bib28] Mak DO, McBride S, Foskett JK (2001). ATP regulation of recombinant type 3 inositol 1,4,5-trisphosphate receptor gating. Journal of General Physiology.

[bib29] Mak DO, White C, Ionescu L, Foskett J, Putney J. W (2005). Nuclear patch clamp electrophysiology of inositol trisphosphate receptor Ca2+ release channel. Calcium Signaling.

[bib30] Mak DO, Pearson JE, Loong KP, Datta S, Fernández-Mongil M, Foskett JK (2007). Rapid ligand-regulated gating kinetics of single inositol 1,4,5-trisphosphate receptor Ca^2+^ release channels. EMBO Reports.

[bib31] Mak DO, Vais H, Cheung KH, Foskett JK (2013a). Isolating nuclei from cultured cells for patch-clamp electrophysiology of intracellular Ca^2+^ channels. Cold Spring Harbor Protocols.

[bib32] Mak DO, Vais H, Cheung KH (2013b). Patch-clamp electrophysiology of intracellular Ca^2+^ channels. Cold Spring Harbor Protocols.

[bib33] Mak DO, Vais H, Cheung KH, Foskett JK (2013c). Nuclear patch-clamp electrophysiology of Ca^2+^ channels. Cold Spring Harbor Protocols.

[bib34] Mak DO, Foskett JK (2015). Inositol 1,4,5-trisphosphate receptors in the endoplasmic reticulum: a single-channel point of view. Cell Calcium.

[bib35] McArthur S, Yazid S, Christian H, Sirha R, Flower R, Buckingham J, Solito E (2009). Annexin A1 regulates hormone exocytosis through a mechanism involving actin reorganization. The FASEB Journal.

[bib36] Mekahli D, Bultynck G, Parys JB, De Smedt H, Missiaen L (2011). Endoplasmic-reticulum calcium depletion and disease. Cold Spring Harbor Perspectives in Biology.

[bib37] Mirkowska P, Hofmann A, Sedek L, Slamova L, Mejstrikova E, Szczepanski T, Schmitz M, Cario G, Stanulla M, Schrappe M, van der Velden VH, Bornhauser BC, Wollscheid B, Bourquin JP (2013). Leukemia surfaceome analysis reveals new disease-associated features. Blood.

[bib38] Newton CL, Mignery GA, Südhof TC (1994). Co-expression in vertebrate tissues and cell lines of multiple inositol 1,4,5-trisphosphate (InsP_3_) receptors with distinct affinities for InsP_3_. The Journal of Biological Chemistry.

[bib39] Paknejad N, Hite RK (2018). Structural basis for the regulation of inositol trisphosphate receptors by Ca^2+^ and IP_3_. Nature Structural & Molecular Biology.

[bib40] Perretti M, Christian H, Wheller SK, Aiello I, Mugridge KG, Morris JF, Flower RJ, Goulding NJ (2000). Annexin I is stored within gelatinase granules of human neutrophil and mobilized on the cell surface upon adhesion but not phagocytosis. Cell Biology International.

[bib41] Qin F, Auerbach A, Sachs F (2000). A direct optimization approach to hidden markov modeling for single channel kinetics. Biophysical Journal.

[bib42] Raynal P, Pollard HB (1994). Annexins: the problem of assessing the biological role for a gene family of multifunctional calcium- and phospholipid-binding proteins. Biochimica Et Biophysica Acta (BBA) - Reviews on Biomembranes.

[bib43] Scannell M, Flanagan MB, deStefani A, Wynne KJ, Cagney G, Godson C, Maderna P (2007). Annexin-1 and peptide derivatives are released by apoptotic cells and stimulate phagocytosis of apoptotic neutrophils by macrophages. The Journal of Immunology.

[bib44] Solito E, Nuti S, Parente L (1994). Dexamethasone-induced translocation of lipocortin (annexin) 1 to the cell membrane of U-937 cells. British Journal of Pharmacology.

[bib45] Stathopulos PB, Zheng L, Li GY, Plevin MJ, Ikura M (2008). Structural and mechanistic insights into STIM1-mediated initiation of store-operated calcium entry. Cell.

[bib46] Stephens M (2017). False discovery rates: a new deal. Biostatistics.

[bib47] Sugawara H, Kurosaki M, Takata M, Kurosaki T (1997). Genetic evidence for involvement of type 1, type 2 and type 3 inositol 1,4,5-trisphosphate receptors in signal transduction through the B-cell antigen receptor. The EMBO Journal.

[bib48] Suzuki J, Kanemaru K, Ishii K, Ohkura M, Okubo Y, Iino M (2014). Imaging intraorganellar Ca^2+^ at subcellular resolution using CEPIA. Nature Communications.

[bib49] Thillaiappan NB, Chavda AP, Tovey SC, Prole DL, Taylor CW (2017). Ca^2+^ signals initiate at immobile IP_3_ receptors adjacent to ER-plasma membrane junctions. Nature Communications.

[bib50] Vais H, Foskett JK, Mak DO (2010a). Unitary Ca^2+ ^current through recombinant type 3 InsP_3 _receptor channels under physiological ionic conditions. Journal of General Physiology.

[bib51] Vais H, Siebert AP, Ma Z, Fernández-Mongil M, Foskett JK, Mak DO (2010b). Redox-regulated heterogeneous thresholds for ligand recruitment among InsP_3_R Ca^2+^-release channels. Biophysical Journal.

[bib52] Vais H, Foskett JK, Ullah G, Pearson JE, Mak DO (2012). Permeant calcium ion feed-through regulation of single inositol 1,4,5-trisphosphate receptor channel gating. Journal of General Physiology.

[bib53] Wojcikiewicz RJ (1995). Type I, II, and III inositol 1,4,5-trisphosphate receptors are unequally susceptible to down-regulation and are expressed in markedly different proportions in different cell types. Journal of Biological Chemistry.

[bib54] Yamasaki-Mann M, Parker I (2011). Enhanced ER Ca^2+^ store filling by overexpression of SERCA2b promotes IP_3_-evoked puffs. Cell Calcium.

[bib55] Yáñez M, Gil-Longo J, Campos-Toimil M (2012). Calcium binding proteins. Advances in Experimental Medicine and Biology.

[bib56] Yoo SH, Lewis MS (2000). Interaction of chromogranin B and the near N-terminal region of chromogranin B with an intraluminal loop peptide of the inositol 1,4, 5-trisphosphate receptor. Journal of Biological Chemistry.

[bib57] Zampese E, Pizzo P (2012). Intracellular organelles in the Saga of Ca^2+^ homeostasis: different molecules for different purposes?. Cellular and Molecular Life Sciences.

