# Supplementary material for: ER-luminal [Ca2+] regulation of InsP3 receptor gating mediated by an ER-luminal peripheral Ca2+-binding protein
Source: eLife. 2020 May 18;9:e53531. doi: 10.7554/eLife.53531 (PMC7259957; doi:10.7554/eLife.53531)
Supplement: Figure 6—source data 1. — The spectral count entry is black when the count ratio (experiment count/control count) is ≥1.2; grey when 1.2 > count ratio≥1.0; and grey italics when count ratio <1.0. lfsr is the local false sign rate (see Statistical analysis of mass spectrometry output in the Materials and methods section). The lfsr ≤0.2 (corresponds to a global false discovery rate ≤5%) are bolded. [file elife-53531-fig6-data1.docx]

| Protein | Accession | Cow | A | Cow | B | Cow | C | Local false sign rate |
| --- | --- | --- | --- | --- | --- | --- | --- | --- |
| identified | number | Exp’t | Control | Exp’t | Control | Exp’t | Control | *lfsr* |
| Anx A1 | P46193 | 158 | 99 | 49 | 17 | 142 | 60 | **0.000** |
| Anx A6 | P79134 | 311 | 207 | 185 | 177 | 457 | 259 | **0.000** |
| Anx A11 | P27214 | 23 | 21 | 34 | 13 | 148 | 77 | **0.000** |
| Anx A4 | P13214 | **35** | **10** | 17 | 16 | 68 | 21 | **0.000** |
| Anx A7 | P20072 | *55* | *63* | 34 | 19 | 95 | 56 | **0.200** |
| Anx A2 | P04272 | 169 | 138 | 121 | 84 | *102* | *103* | 0.327 |
| Anx A3 | F1MWQ2 | 37 | 17 | 7 | 1 | *1* | *2* | 0.647 |
| Anx A13 | F1N2Q7 | 2 | 0 | 0 | 0 | 0 | 0 | 0.918 |
| Anx A5 | P81287 | 4 | 3 | 0 | 0 | 0 | 0 | 0.929 |

**Figure 6-source data 1. Abundance of annexin proteins (quantified as total spectral counts by mass spectrometry analysis) detected in experiment eluates collected from magnetic beads covalently linked to peptides with modified pL2 sequence, and in control eluates collected from beads linked to peptide with scrambled modified pL2 sequence.** The spectral count entry is black when the count ratio (experiment count/control count) is ≥ 1.2; grey when 1.2 > count ratio ≥ 1.0; and grey italics when count ratio < 1.0. *lfsr* is the local false sign rate (see *Statistical analysis of mass spectrometry output* in the Materials and Methods section). The *lfsr* ≤ 0.2 (corresponds to a global false discovery rate ≤ 5%) are bolded.

_________________________________________________________________________________
