# Supplementary material for: ER-luminal [Ca2+] regulation of InsP3 receptor gating mediated by an ER-luminal peripheral Ca2+-binding protein
Source: eLife. 2020 May 18;9:e53531. doi: 10.7554/eLife.53531 (PMC7259957; doi:10.7554/eLife.53531)
Supplement: Transparent reporting form [file elife-53531-transrepform.docx]

***eLife’s* transparent reporting form**

We encourage authors to provide detailed information *within their submission* to facilitate the interpretation and replication of experiments. Authors can upload supporting documentation to indicate the use of appropriate reporting guidelines for health-related research (see [EQUATOR Network](http://www.equator-network.org/%20)), life science research (see the [BioSharing Information Resource](https://biosharing.org/)), or the [ARRIVE guidelines](http://www.plosbiology.org/article/info:doi/10.1371/journal.pbio.1000412) for reporting work involving animal research. Where applicable, authors should refer to any relevant reporting standards documents in this form.

If you have any questions, please consult our Journal Policies and/or contact us: [editorial@elifesciences.org](mailto:editorial@elifesciences.org).

**Sample-size estimation**

- You should state whether an appropriate sample size was computed when the study was being designed
- You should state the statistical method of sample size computation and any required assumptions
- If no explicit power analysis was used, you should describe how you decided what sample (replicate) size (number) to use

Please outline where this information can be found within the submission (e.g., sections or figure legends), or explain why this information doesn’t apply to your submission:

Because our experiments were done exclusively using cell lines, with no animal involved. Therefore, we have not performed any power analysis.

We determined our sample size based on the amount of time we can reasonably perform the experiments due to technical limitation, not based on a target sample size determined when the study was designed.

**Replicates**

- You should report how often each experiment was performed
- You should include a definition of biological versus technical replication
- The data obtained should be provided and sufficient information should be provided to indicate the number of independent biological and/or technical replicates
- If you encountered any outliers, you should describe how these were handled
- Criteria for exclusion/inclusion of data should be clearly stated
- High-throughput sequence data should be uploaded before submission, with a private link for reviewers provided (these are available from both GEO and ArrayExpress)

Please outline where this information can be found within the submission (e.g., sections or figure legends), or explain why this information doesn’t apply to your submission:

The number of replicate performed in each experiment was indicated in the figures in the main text and the supplementary figures.

Since we used exclusively cell lines in our experiments, we consider each experiment as a technical replicate only.

In our global calcium fluorescence experiment of intact cells stimulated with histamine, the criteria for outliner exclusion is clearly described in the Methods and Material section p. 40-41, line 788-815 in the manuscript.

In all patch-clamp experiments, single-channel current traces that were too noisy due to poor seal formation or baseline instability to be properly analyzed were excluded.

We did not perform any high-throughput sequencing.

**Statistical reporting**

- Statistical analysis methods should be described and justified
- Raw data should be presented in figures whenever informative to do so (typically when N per group is less than 10)
- For each experiment, you should identify the statistical tests used, exact values of N, definitions of center, methods of multiple test correction, and dispersion and precision measures (e.g., mean, median, SD, SEM, confidence intervals; and, for the major substantive results, a measure of effect size (e.g., Pearson's r, Cohen's d)
- Report exact p-values wherever possible alongside the summary statistics and 95% confidence intervals. These should be reported for all key questions and not only when the p-value is less than 0.05.

Please outline where this information can be found within the submission (e.g., sections or figure legends), or explain why this information doesn’t apply to your submission:

We described our statistical methods used in the Methods and Materials section and/or figure legends pertaining to individual experiments.

Raw data were clearly marked (as dots) in each individual Figure whenever that is appropriate.

Mean and s.e.m. are indicated by symbols and error bars next to the raw data dots in every figure.

Values of N are tabulated on top of the mean and s.e.m. indicators in every figure.

P values of all significant t-tests (p value < 0.05) are tabulated in every figures.

(For large datasets, or papers with a very large number of statistical tests, you may upload a single table file with tests, Ns, etc., with reference to sections in the manuscript.)

**Group allocation**

- Indicate how samples were allocated into experimental groups (in the case of clinical studies, please specify allocation to treatment method); if randomization was used, please also state if restricted randomization was applied
- Indicate if masking was used during group allocation, data collection and/or data analysis

Please outline where this information can be found within the submission (e.g., sections or figure legends), or explain why this information doesn’t apply to your submission:

Not applicable

**Additional data files (“source data”)**

- We encourage you to upload relevant additional data files, such as numerical data that are represented as a graph in a figure, or as a summary table
- Where provided, these should be in the most useful format, and they can be uploaded as “Source data” files linked to a main figure or table
- Include model definition files including the full list of parameters used
- Include code used for data analysis (e.g., R, MatLab)
- Avoid stating that data files are “available upon request”

Please indicate the figures or tables for which source data files have been provided:

Source data files of the following Figures are provided:

Fig 2I&J and Supplementary Fig S1.xlsx

Fig 3G&H.xlsx

Fig 4J.xlsx

Fig 5E.xlsx

Fig 6F, Fig 8 and Supplementary Fig. S3&4.xlsx

Fig 6L.xls

Fig 7C & Fig 8.xlsx

Fig 9B + or - AnxA1.xls

Fig 9B.xlsx

Fig 9E.xlsx

Fig 10C-E.xlsx

Fig 10G&J.xlsx

Fig 10H.xlsx

Fig 10I.xlsx

Fig 10K.xlsx

Fig 10M&N.xlsx

Fig 10O&P.xlsx

t-test result for Figures.xlsx

mean, sem, N of data in Figures.xlsx
